# Input and Output Connections of the Crow Nidopallium Caudolaterale

**DOI:** 10.1523/ENEURO.0098-24.2024

**Published:** 2024-04-18

**Authors:** Ylva Kersten, Felix W. Moll, Saskia Erdle, Andreas Nieder

**Affiliations:** Animal Physiology Unit, Institute of Neurobiology, University of Tübingen, Tübingen 72076, Germany

**Keywords:** avian brain, cholera toxin, corvid songbird, dextran amine, pallium, telencephalon, tract-tracing

## Abstract

The avian telencephalic structure nidopallium caudolaterale (NCL) functions as an analog to the mammalian prefrontal cortex. In crows, corvid songbirds, it plays a crucial role in higher cognitive and executive functions. These functions rely on the NCL's extensive telencephalic connections. However, systematic investigations into the brain-wide connectivity of the NCL in crows or other songbirds are lacking. Here, we studied its input and output connections by injecting retrograde and anterograde tracers into the carrion crow NCL. Our results, mapped onto a published carrion crow brain atlas, confirm NCL multisensory connections and extend prior pigeon findings by identifying a novel input from the hippocampal formation. Furthermore, we analyze crow NCL efferent projections to the arcopallium and report newly identified arcopallial neurons projecting bilaterally to the NCL. These findings help to clarify the role of the NCL as central executive hub in the corvid songbird brain.

## Significance Statement

The cognitive abilities of corvid songbirds are associated with the nidopallium caudolaterale (NCL), considered a functional analog of the mammalian prefrontal cortex. However, the connectivity of the NCL in corvids, or any songbird, with the rest of the brain has remained unknown. We investigated the input and output connections of the carrion crow NCL by using retrograde and anterograde tracers. Our results confirm the existence of multisensory connections to the NCL. Additionally, we examined the efferent projections from the NCL to the arcopallium and identified previously unknown arcopallial neurons that project bilaterally to the NCL. These findings contribute to a better understanding of the role of the NCL as a central executive hub in the corvid brain.

## Introduction

Avian and mammalian endbrains differ radically in their overall organization, reflecting >300 million years of independent evolution (Karten, 2015; Striedter, 2016; Stacho et al., 2020). In both groups, the number of associative endbrain neurons appears to be a proxy for cognitive complexity (Herculano-Houzel, 2009, 2011; Kverková et al., 2022). However, while these neurons populate the layered neocortex emerging from the embryonic dorsal pallium in mammals, they form the structurally and ontogenetically distinct dorsal ventricular ridge (DVR) that arises from the ventral pallium in birds (Reiner et al., 2004b; Jarvis et al., 2005; Karten, 2015; Güntürkün and Bugnyar, 2016).

One crucial component of the DVR is the nidopallium caudolaterale (NCL), which sits at the pallial interface of ascending, multisensory inputs and descending premotor outputs (Kröner and Güntürkün, 1999; Farries, 2001; Fernández et al., 2020b; Zemel et al., 2023). Consistent with its multimodal connectivity, the NCL plays a central role in various higher cognitive functions such as working memory (Veit et al., 2014; Moll and Nieder, 2017), motor planning (Rose and Colombo, 2005; Rinnert and Nieder, 2021; Kirschhock and Nieder, 2022; Hahn and Rose, 2023), prospective problem-solving (Veit and Nieder, 2013; Moll and Nieder, 2015; Veit et al., 2015b), magnitude estimation (Moll and Nieder, 2014; Ditz and Nieder, 2015; Kirschhock and Nieder, 2023; Wagener and Nieder, 2023), spatial cognition (Veit et al., 2015a; Rinnert et al., 2019), or sensory consciousness (Nieder et al., 2020). Moreover, several studies in pigeons have shown that inactivation of the NCL can affect a subset of the aforementioned capacities, highlighting its critical role in behavioral flexibility (Mogensen and Divac, 1982, 1993; Gagliardo et al., 1996, 1997; Güntürkün, 1997; Hartmann and Güntürkün, 1998; Helduser and Güntürkün, 2012). Thus, the existing evidence supports the idea that the avian NCL serves as a functional equivalent of the mammalian PFC (Güntürkün, 2005; Moll and Nieder, 2015; Nieder, 2017, 2023).

The extent of the NCL is typically estimated based on an immunohistochemical staining method against tyrosine hydroxylase (TH), the rate-limiting enzyme in the production of dopamine (Divac and Mogensen, 1985; Kröner and Güntürkün, 1999). These midbrain, dopaminergic afferents are denser within the NCL compared with neighboring nidopallial areas, which signifies another similarity to the PFC (Divac and Mogensen, 1985; von Eugen et al., 2020; Kersten et al., 2022). Notably, the anti-TH method identifies a single portion of the pigeon caudal nidopallium, whereas it distinguishes three caudal nidopallial areas in the carrion crow and other songbirds (i.e., a dorsal, medial, and ventral NCL; von Eugen et al., 2020; Kersten et al., 2022). Given its position relative to the readily identifiable dorsal arcopallial tract (DA), the projections of the crow's dorsal NCL (NCLd) to the arcopallium appear to correspond to the arcopallial projections of the NCL in pigeons (von Eugen et al., 2020; Kersten et al., 2022). Therefore, comparative functional studies were focused on investigating the crow's NCLd over the past decade (Veit and Nieder, 2013; Kersten et al., 2022; Wagener and Nieder, 2023). However, the brain-wide connectivity of the NCLd in the crow, or any other songbird, has not yet been explored systematically (Wild and Farabaugh, 1996; Paterson and Bottjer, 2017).

The NCLd is part of the nidopallium, which is exceptionally large in corvid songbirds, such as crows, jays, jackdaws, magpies, and ravens (Iwaniuk and Hurd, 2005; Mehlhorn et al., 2010). Although hyperplasia (Kubke et al., 2004) has not been demonstrated for the corvid nidopallium, the absolute number of nidopallial neurons in carrion crows is approximately nine times higher compared with that in pigeons or chickens (Rehkämper et al., 1991; Ströckens et al., 2022). Thus, it has been suggested that the sheer number of associative neurons could be a driver of advanced corvid behaviors such as tool use or future planning (Hunt, 1996; Bird and Emery, 2009; Kabadayi and Osvath, 2017; Ströckens et al., 2022). Alternatively, or in addition, connectivity differences may exist between associative structures in corvids and nonpasserine birds. Here we present an initial effort to answer this question by focusing on the functionally well-characterized example of the carrion crow NCLd (Nieder, 2017). We find that its brain-wide connectivity is similar to the pigeon NCL and thus potentially conserved across avian taxa.

## Materials and Methods

### Animals

We used three hand-raised adult male carrion crows (*Corvus corone*; age range, 8–12 years) obtained from the Institutes’ breeding stock. The crows had lived in spacious aviaries in captivity throughout their lives (Hoffmann et al., 2011). All crows had participated in combined behavioral–electrophysiological experiments and were fully healthy prior to the histological investigations. The crows’ body weights ranged between 490 and 635 g with rostrocaudal dimensions of the crows’ telencephala between 21.6 and 23.3 mm and brain weights from 7.1 to 7.8 g (measured postperfusion). All procedures were carried out according to the guidelines for animal experimentation and approved by the responsible national authorities, the Regierungspräsidium Tübingen, Germany.

### Surgical procedures

All surgeries were performed while the animals were under general anesthesia. Crows were anaesthetized with a ketamine/xylazine mixture (50 mg ketamine, 5 mg/kg xylazine initially, supplemented by smaller dosages in regular intervals on demand) and received analgesics postsurgically (Ditz and Nieder, 2016). During anesthesia, the head was placed in a commercially available stereotactic holder (David Kopf Instruments, Model 1430 Stereotaxic Frame) and ear bars for pigeons (Model 856 Ear Bars; 20° tapered tip to a 4.8 mm shoulder with a 3 mm dia. by 2-mm-long protrusion). A simple beak biting rod was added so that the beak would be held in a 45° angle below the horizontal axis. Based on previously described coordinates (Kersten et al., 2022), NCLd was accessed through a small craniotomy centered at 3.5 mm posterior and 11.5 mm lateral relative to the center of the bifurcation of the superior sagittal sinus. This AP position corresponds to “AP 4.6” in our previously published carrion crow brain atlas (Kersten et al., 2022).

### *In vivo* stereotaxic injections

We used glass pipettes (opening diameter, 20 μm) with an oil-based pressure injection system (Nanoject III, Drummond Scientific) for all tracer injections. Each hemisphere was injected at three mediolateral injection sites (ML 10.5, 11.8, and 12.5 mm) along one fixed AP value ranging from -3.65 to -2.9 mm relative to the center of the bifurcation of the superior sagittal sinus and 1.0 mm below the surface of the brain at a 90° injection angle (i.e., perpendicular to the horizontal plane). We targeted one fixed AP value in a given hemisphere but varied this value at few individual injection sites by a maximum of ±0.2 mm to avoid blood vessel collisions. Individual hemispheres were either injected with a retrograde or anterograde tracer. We used cholera toxin subunit B (CTB, Invitrogen, Alexa Fluor 488 or 555 conjugate, C34775 or C34776, respectively) as a retrograde tracer and injected 200 nl CTB per site (1.0%, diluted in physiological saline solution; 1 nl pulses at 0.5 Hz with an injection speed of 20 nl/s, total injection time per site: 400 s, after the last injection pulse the injection needle was initially left in place for 10 min prior to its retraction). To visualize anterograde projection targets, we used dextran amine conjugates (10,000 MW, Invitrogen, Alexa Fluor 488 D22910, or fluoro Ruby D1817, 50 mg/ml, diluted in physiological saline solution) and injected 200–300 nl per site.

### Histology

Ten days after the tracer injections, crows were injected (i.m.) with 0.5 ml of heparin (Braun, 100,000 I.E./10 ml) and a lethal dosage of sodium pentobarbital (Boehringer Ingelheim, Narcoren, 2.5 ml/kg). Subsequently, we perfused the birds with 0.12 M phosphate-buffered saline (PBS) including 0.1% heparin, followed by 4% paraformaldehyde (PFA) in 0.12 M phosphate buffer (PB). The brains were removed from the skull and postfixed in 4% PFA overnight. Next, they were sunk in an uprising sucrose solution, ending in 30% sucrose solution. Hemispheres were cut at 50 µm using a cryostat (Leica Biosystems, CM1900) in sagittal or coronal orientation. Slices were collected in PB buffer and stored in antifreeze solution, containing glycerol and ethylene glycol, and stored at −20°C. We mounted series of these slices on SuperFrost Ultra Plus object plates (Thermo Fisher Scientific) and covered them with Vectashield antifade mounting medium including DAPI (H-1200 Vector Laboratories). In addition, we Nissl stained a subset of slices as previously described (Kersten et al., 2021). In short, slides were incubated in a warm (55°C) 0.1% cresyl violet solution for 3 min, washed in 0.012 M PB, and dehydrated and differentiated in an uprising ethanol series. After immersing them in xylene, they were mounted with Entellan mounting medium (Merck). We imaged using a Leica (DMi8) epifluorescence microscope and, to demonstrate the colocalization of somata and axons in Figure 7*c*, a Zeiss (Airyscan 2) confocal microscope. Image analysis was performed using Fiji (Schindelin et al., 2012) and ZEN (Zen 2.5 lite, blue edition, Carl Zeiss) software. To determine the exact position of individual dye labeled cells relative to established brain structures (Kersten et al., 2022), we used polarized light to identify fiber tracts and laminae within slice. Additionally, directly adjacent, Nissl stained slices were used to visualize brain nuclei. We then used this information to manually register individual cells with atlas drawings generated in Corel DRAW X7 (Kersten et al., 2022). When graphically indicated, the extent of the NCLd (i.e., the target area of our injections) represents a conservative estimate, which is based on our previously published staining against TH (Kersten et al., 2022).

## Results

### General results

**Figure 1.**
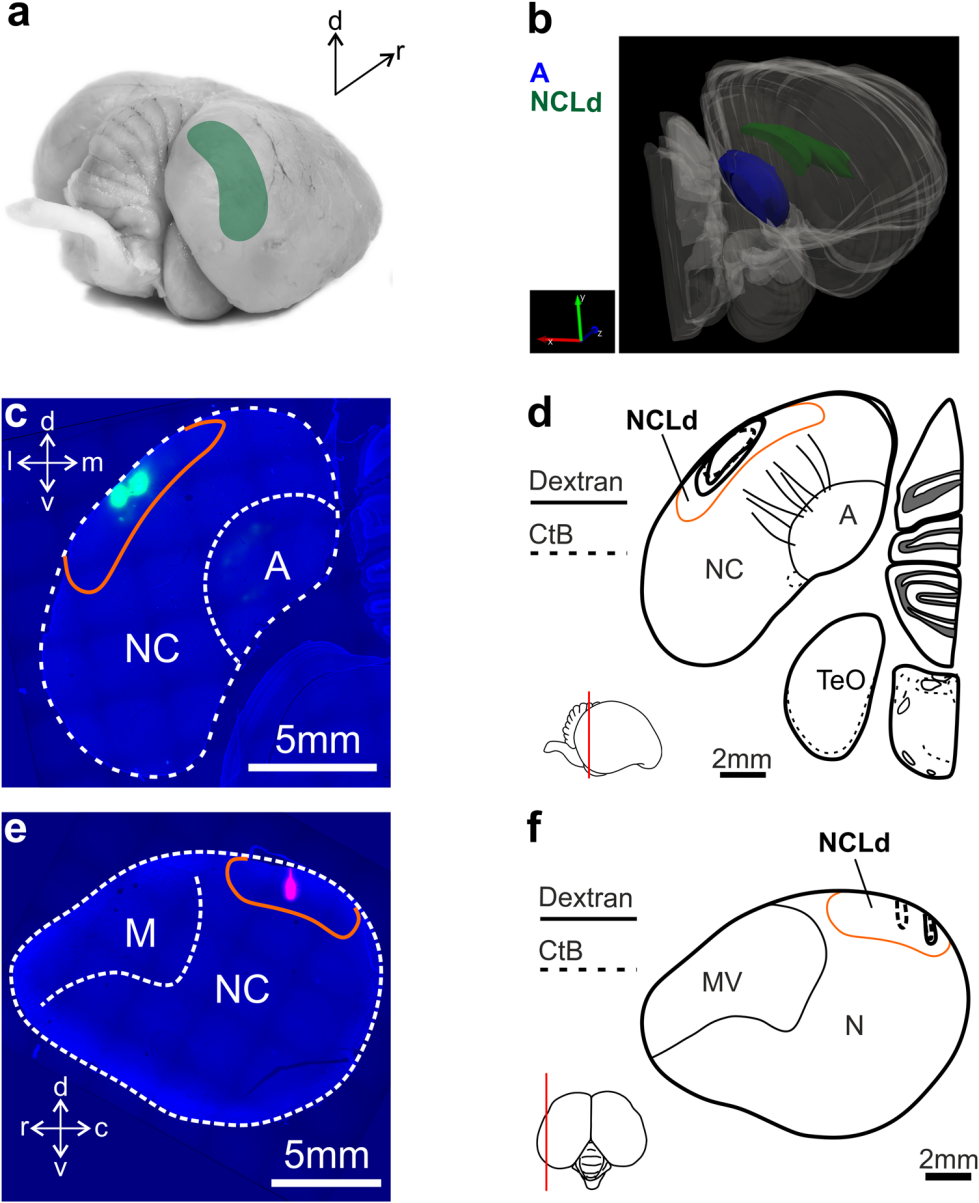
Injection sites within the crow NCLd. We injected retrograde (CTB) or anterograde (dextran) tracers into the NCLd of the carrion crow. ***a***, Photography of the carrion crow brain. The shading marks the location of the NCLd in the crow's posterior telencephalon. ***b***, Three-dimensional reconstruction of the right hemisphere showing the NCLd in green and, as a reference, the arcopallium in blue (based on Kersten et al., 2022). Injection sites (*n* = 6 hemispheres in 3 birds) were confirmed by histological analysis of either coronal (***c***,***d***) or sagittal slices (***e***,***f***). ***c***,***e***, Exemplary brain tissue slices cutting through the center of two of three injection boluses in a coronal slice (***c***) (the third most medial bolus appears much fainter as the AP position of individual injections varied slightly to avoid blood vessel collisions; also see Materials and Methods) and through one of three boluses in the sagittal slice (***e***). The white dotted line outlines the telencephalon and other references structures. NCLd is indicated by the orange line. Green (***c***) and magenta (***e***) colors indicate the dye spread. ***d***,***f*** Show the outline of all three injection sites for each hemisphere projected onto a single, schematic coronal (***d***) or sagittal (***f***) plane. Scale bars: ***c***,***e***, 5 mm; ***d***,***e***, 2 mm.

**Figure 2.**
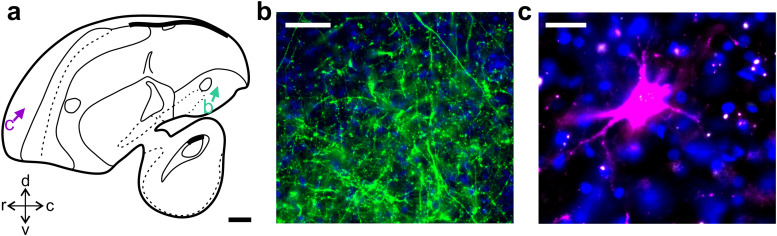
Dye coupled tracers robustly label crow brain structures. ***a***, Schematic of the crow brain (sagittal view) with arrows pointing to neuronal structures detailed in ***b*** and ***c***. ***b***, Microscopic image (epifluorescence; *z* stack projection) of a terminal field of anterogradely labeled NCLd axons (green) within the arcopallium. ***c***, A retrogradely labeled NCLd-projecting neuron (magenta) from the rostral hyperpallium. DAPI stained nuclei are shown in blue (***b***,***c***). Scale bars: ***a***, 2 mm; ***b***, 50 µm; ***c***, 20 µm.

To identify monosynaptic afferent inputs and efferent projections of the carrion crow NCLd (Fig. 1*a*,*b*), we injected retrograde or anterograde fluorescent dye coupled tracers (CTB or dextran amines, respectively) into the NCLd (Kersten et al., 2022) of three carrion crows (Table 1). Histological analysis confirmed that all our injection sites were located in close proximity to the center of mass of NCLd (*n* = 6 hemispheres in 3 birds; Fig. 1*c–f*). Similar to previous songbird studies that utilized fluorescent dye coupled dextran amines or CTB (Paterson and Bottjer, 2017; Düring et al., 2020), we observed a high and robust signal-to-noise ratio of labeled fibers and somata (Fig. 2). Collectively, we found retrogradely CTB labeled somata in both telencephalic and subtelencephalic areas of the brain and across hemispheres (*n* = 3 hemispheres in 2 birds), whereas anterogradely dextran amine labeled axons appeared to be restricted to the ipsilateral telencephalon (*n* = 3 hemispheres in 2 birds). All our findings were consistently replicated in three hemispheres across two birds, if not stated otherwise.

**Table 1.** Detailed injection protocol

Animal	Injections		Slicing	
	Left hemisphere	Right hemisphere	Left hemisphere	Right hemisphere
Crow 1	D (555 nm)	D (488 nm)	Coronal	Coronal
Crow 2	CTB (555 nm)	CTB (488 nm)	Sagittal	Coronal
Crow 3	CTB (555 nm)	D (488 nm)	Sagittal	Sagittal

**Figure 3.**
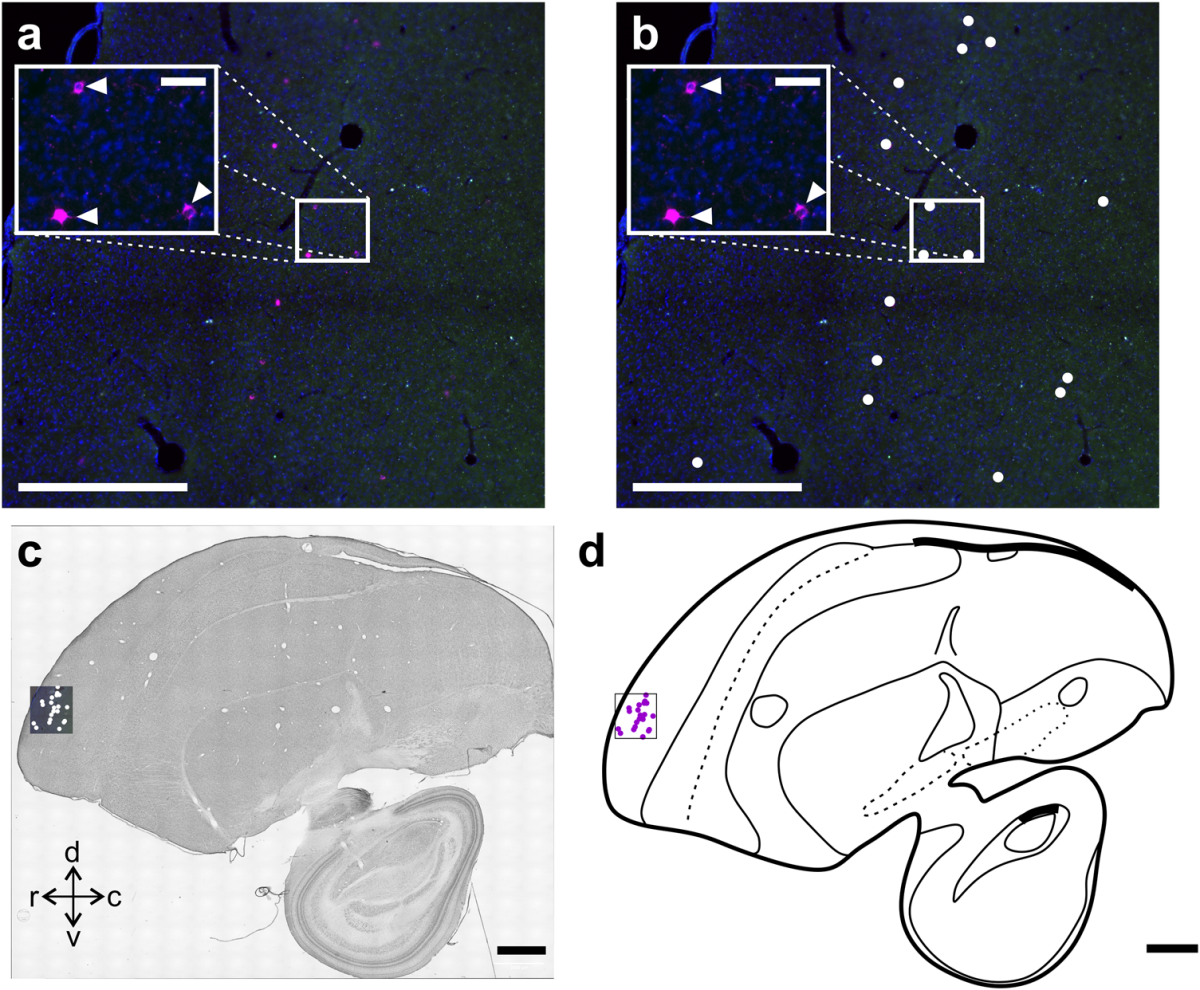
Brain-wide registration of labeled somata. Step-by-step description of how we registered retrogradely labeled neurons to an existing atlas of the carrion crow brain (Kersten et al., 2022). ***a***, ***b***, The positions of labeled somata were identified in high-resolution images and marked with a dot symbol. ***c***, Next, the positions of labeled somata were registered to directly adjacent Nissl stained slices. ***d***, Last, we used the structural information of the Nissl stained slices to generate line drawings that are based on a stereotactic brain atlas of the carrion crow (Kersten et al., 2022). Scale bars: ***a***,***b***, 500 µm; ***c***,***d***, 2 mm.

**Figure 4.**
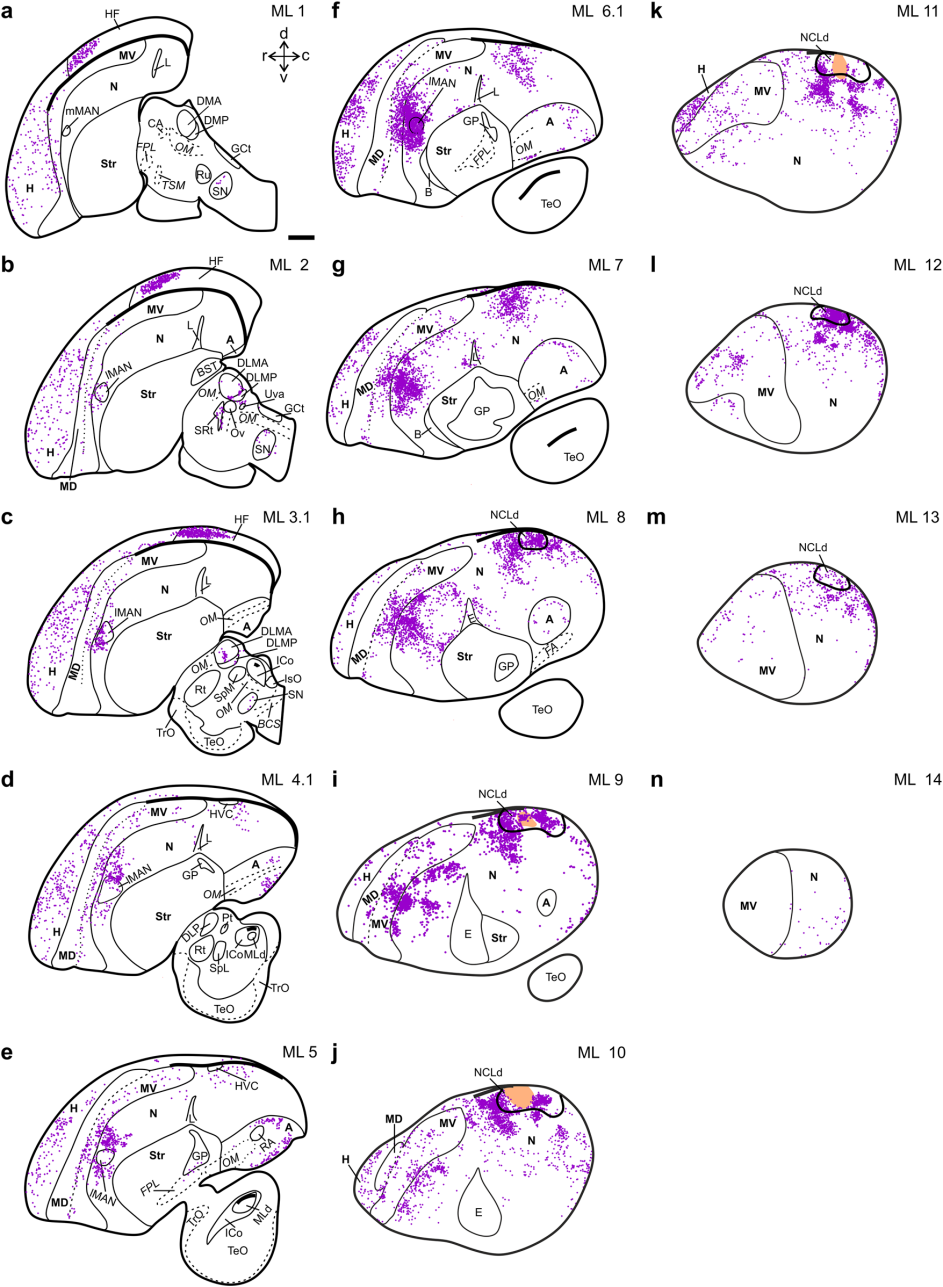
NCLd-projecting neurons throughout the crow brain. A complete mapping of one representative crow brain (sagittal view) with each dot representing a cell retrogradely labeled by our NCLd injection. The distance (in mm) from the midline is noted at the top right of each section (***a–n***). The orange areas in ***i–k*** mark the injection sites and the extent of the tracer spread. A, arcopallium; ALp, posterior nucleus of the ansa lenticularis; B, nucleus basorostralis pallii; CA, commissura anterior; DL, dorsolateral nucleus of the thalamus; DLMA, medial part of the dorsolateral nucleus of the anterior thalamus, anterior part; DLMP, medial part of the dorsolateral nucleus of the anterior thalamus, posterior part; DMA, nucleus doesomedialis anterior thalami; DMP, nucleus doesomedialis posterior thalami; E, entopallium; FPL, lateral prosencephalic fascicle; GCt, substantia grisea centralis; GP, globus pallidus; H, hyperpallium; HF, hippocampal formation; HL, nucleus habenularis lateralis; HM, nucleus habenularis medialis; HVC, high vocal center; ICo, nucleus intercollicularis; IPo, intermedioposterior nucleus; IsO, isthmooptic nucleus; L, field L; lMAN, lateral part of the magnocellular nucleus of the anterior nidopallium; mMAN, medial part of the magnocellular nucleus of the anterior nidopallium; MD, dorsal mesopallium; MLd, nucleus mesencephalicus lateralis; MV, ventral mesopallium; N, nidopallium; NCLd, dorsal part of the nidopallium caudolaterale; OM, occipito-mesencephalic tract; Ov, nucleus ovoidalis; RA, robust nucleus of the arcopallium; Rt, nucleus rotundus; Ru, nucleus ruber; SN, substantia niger; SpL, nucleus spiriformis lateralis; SpM, nucleus spiriformis medialis; SRt, nucleus subrotundus; Str, striatum; TeO, tectum opticum; TrO, tractus opticus; TSM, tractus septopallio-mesenceohalicus; Uva, nucleus uvaeformis; VTA, ventral tegmental area. Scale bars: 2 mm.

To identify afferent and efferent NCLd connections across the entire brain, we examined equally spaced full brain slices (interslice spacing, 150 µm) from all hemispheres. High-resolution images from the left hemisphere of one representative bird (Crow 2; spacing, 1 mm) were used to comprehensively identify the position of individual, retrogradely labeled somata (Fig. 3*a*,*b*). Somata positions were then manually registered to line drawings of each inspected brain slice (Fig. 3*c*,*d*; see Materials and Methods), which are based on a stereotactic atlas of the carrion crow brain (Kersten et al., 2022). We describe the individual brain subdivisions depicted in this retrograde connectivity atlas below (Fig. 4). Additionally, we report brain areas that contained anterogradely labeled axons and their terminal arborizations but refrained from a detailed brain-wide illustration of individual axonal fragments because they were often scattered and rarely observed in dense terminal fields. As an exception to this rule, we did find dense terminal fields within the arcopallium (A) and elaborate on this prominent projection below.

### Hyperpallium and hippocampus

**Figure 5.**
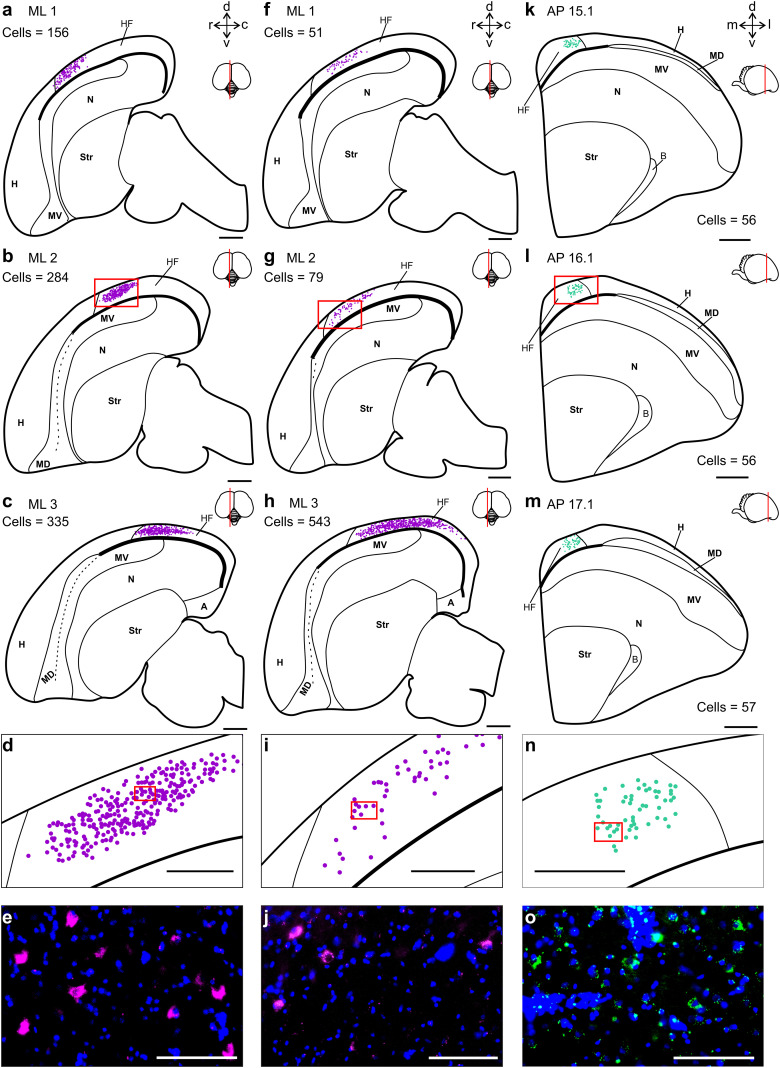
Retrograde CTB labeling in the hippocampal formation across crows. ***a–c***, Distribution of CTB labeled cells (represented by magenta dots) within the hippocampal formation (HF) in three different sagittal slices (ML 1–3) from the left hemisphere of Crow 2 (the same slices are shown in Fig. 4*a–c*). ***d***, A closer view of the labeled cluster of cells shown within the red rectangle in ***b***. ***e***, Histological image of the cells represented by the dots shown within the red rectangle in ***d***, note that a subset of these cells is located off the focal plane. ***f–j***, Same as ***a–e*** for the left hemisphere of Crow 3. ***k–o***, Same as ***a–e*** but for coronal slices from the right hemisphere of Crow 2 (AP 15.1–17.1; coordinates given as in Kersten et al., 2022).

Retrogradely labeled somata were present throughout the entire mediolateral extent of the hyperpallium (H). Those cells were located in the rostral, somatosensory, as well as in the caudal visual aspects of the H (Atoji and Wild, 2019; Stacho et al., 2020; Fig. 4*a–k*). Just caudal to H, we found a distinct and dense cluster of retrogradely labeled cells within the anterior half of the hippocampal formation (Atoji and Wild, 2006; Kersten et al., 2022), which did not extend beyond ML 4.0 in all injected hemispheres (Figs. 4*a–c*, 5; *n* = 3 hemispheres in 2 birds). Interestingly, this hippocampal labeling has not been described in previous tracing studies, in which similar tracers were injected into the dorsolateral nidopallium of pigeons or zebra finches (Leutgeb et al., 1996; Kröner and Güntürkün, 1999; Paterson and Bottjer, 2017).

### Mesopallium

The mesopallium (M) is composed of a dorsal (MD) and a ventral part (MV), separated by the intermediate mesopallial lamina (LMI; Fig. 4; Jarvis et al., 2013; Kersten et al., 2022). Our study revealed retrogradely labeled somata throughout the entire mediolateral extent of MV. The majority of these cells were concentrated in the central region of MV's anterior–posterior axis (Fig. 4*d–h*), which is a mesopallial subdivision that has been shown to receive secondary visual and multisensory information (Atoji and Wild, 2012). In contrast to MV, labeled cells were scarce in MD and primarily located along its borders, specifically the intermediate or the dorsal mesopallial lamina (LMI and LMV; Fig. 4*b–j*). This overall pattern is consistent with the previously described observation in pigeons that MD projects to the central but not dorsolateral nidopallium and the notion that MD and MV are functionally distinct structures (Karten et al., 1973; Atoji and Wild, 2009, 2012; Jarvis et al., 2013).

### Nidopallium

Our injection sites within NCLd were surrounded by many retrogradely labeled cells (Fig. 4*i–k*). These cells formed a continuous cluster that extended throughout the NCLd and beyond the immediate vicinity of our injection sites, indicating rich local connectivity (Fig. 4*d–m*). The cluster stopped short of the most lateral pole of the dorsal NC, consistent with the established lateral border of NCLd (von Eugen et al., 2020; Kersten et al., 2022; Fig. 4*h–m*). At this lateral NC pole, some labeled cells were found in the ventral half of the NCL and, therefore, appeared to exist within the recently described ventral NCL (NCLv; Fig. 4*n*; von Eugen et al., 2020; Kersten et al., 2022). Toward medial, the cluster continuously shifted anterior, while it remained approximately equidistant to the posterior border of the mesopallium (Fig. 4*d–h*). Further medial, the cluster did not exceed the HVC and thus we found no cells in the caudomedial nidopallium (NCM). Similarly, labeled cells were absent in the medial NCL (NCLm), which lines the dorsal border of the arcopallium (Fig. 4*c–g*; von Eugen et al., 2020; Kersten et al., 2022). Taken together, the distribution of labeled cells within NC supports the idea that the crow NCLd is a distinct and highly interconnected subdivision of the NC.

Anterior to the NC, inside the intermediate nidopallium (NI), we found a prominent group of labeled cells concentrated within and close to the shell area of lMAN (Fig. 4*b–f*). Interestingly, the density and extent of the lMAN related NI cluster appeared to be continuous with the neighboring mesopallial cluster of NCLd-projecting MV cells (Fig. 4*b–j*). Together, these two areas have been described as the medial nidopallium/mesopallium (MNM; Hahn and Rose, 2023), which is a reciprocally connected, central hub of the tectofugal system with direct projections to premotor structures of the arcopallium (Atoji and Wild, 2012). Therefore, this area is well positioned to support the executive function of the crow NCLd (Nieder, 2017).

Our data suggest that the crow NCLd receives visual and somatosensory input via the MNM (Wild and Farabaugh, 1996; Sadananda and Bischof, 2006; Atoji and Wild, 2012; Wild and Gaede, 2016). In addition, we observed consistent nidopallial labeling in field L (but not in the thalamorecipient field L2a; Fig. 4*d–g*) and in the periphery of HVC including its shelf region (Fig. 4*d*,*e*), which are collectively a likely source of auditory information to the NCLd (Mello and Clayton, 1994; Vates et al., 1996; Mello et al., 1998). Thus, the nidopallial connections of the NCLd alone are sufficient to make it a genuinely multimodal structure in the carrion crow brain.

### Arcopallium

**Figure 6.**
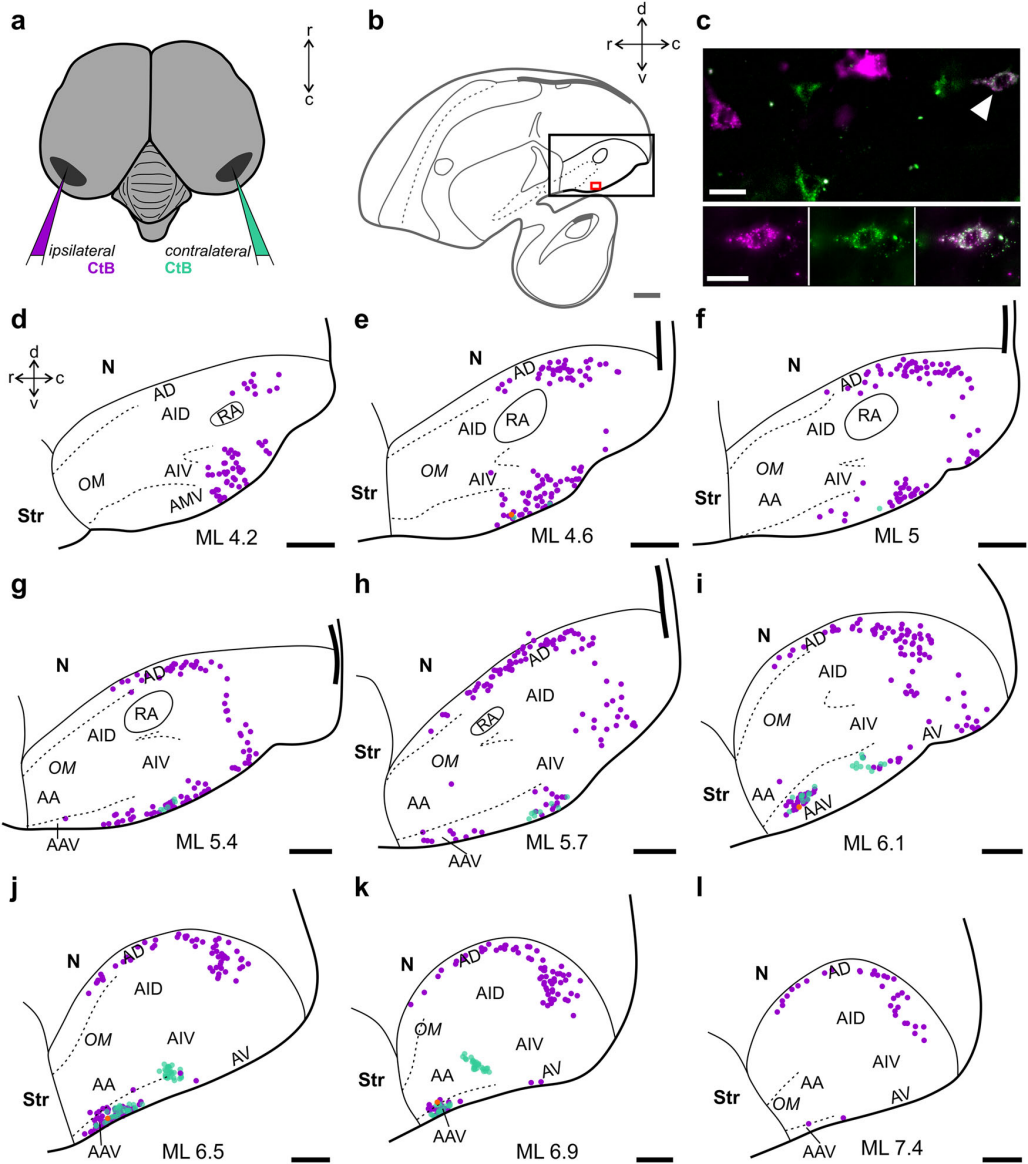
The arcopallium bilaterally projects to the NCLd. ***a***, Arcopallial cells were labeled by retrograde tracers (CTB) from both hemispheres, following bilateral NCLd injections. ***b***, Schematic of the crow brain (sagittal view). The black box indicates the position of the arcopallium. The red box shows the position of a ventral arcopallial area detailed in ***c***. ***c***, Many arcopallial cells projected either ipsi- or contralaterally (magenta or green, respectively). The white arrow indicates a cell that had taken up dye from both hemispheres. This cell is visualized in the lower panel in (from left to right) the magenta or green channel only and with both channels merged. ***d–l***, Equally spaced sagittal line drawings of the arcopallium. Somata that were labeled with ipsilaterally injected dye are depicted in magenta while somata that took up dye from the contralateral hemisphere are depicted in green. Somata that took up dye from both hemispheres are indicated by orange dots. Note that contralaterally projecting somata are restricted to the ventral parts of A (AA, AAV, AV, AIV), whereas ipsilaterally projecting cells are distributed in a dichotomic manner in dorsal (AD) as well as in ventral (AA, AAV, AV, AIV) aspects of A. Double labeled somata also appear in the ventral A (AAV). A, arcopallium; AA, anterior arcopallium; AAV, anterior ventral arcopallium; AD, dorsal arcopallium; AID, dorsal intermediate arcopallium; AIV, ventral intermediate arcopallium; AMV, medial ventral arcopallium; AV, ventral Arcopallium; OM, occipito-mesencephalic tract; Str, striatum. Scale bars: ***b***, 2 mm; ***c***, 20 µm; ***d–l*** vb, 1 mm.

**Figure 7.**
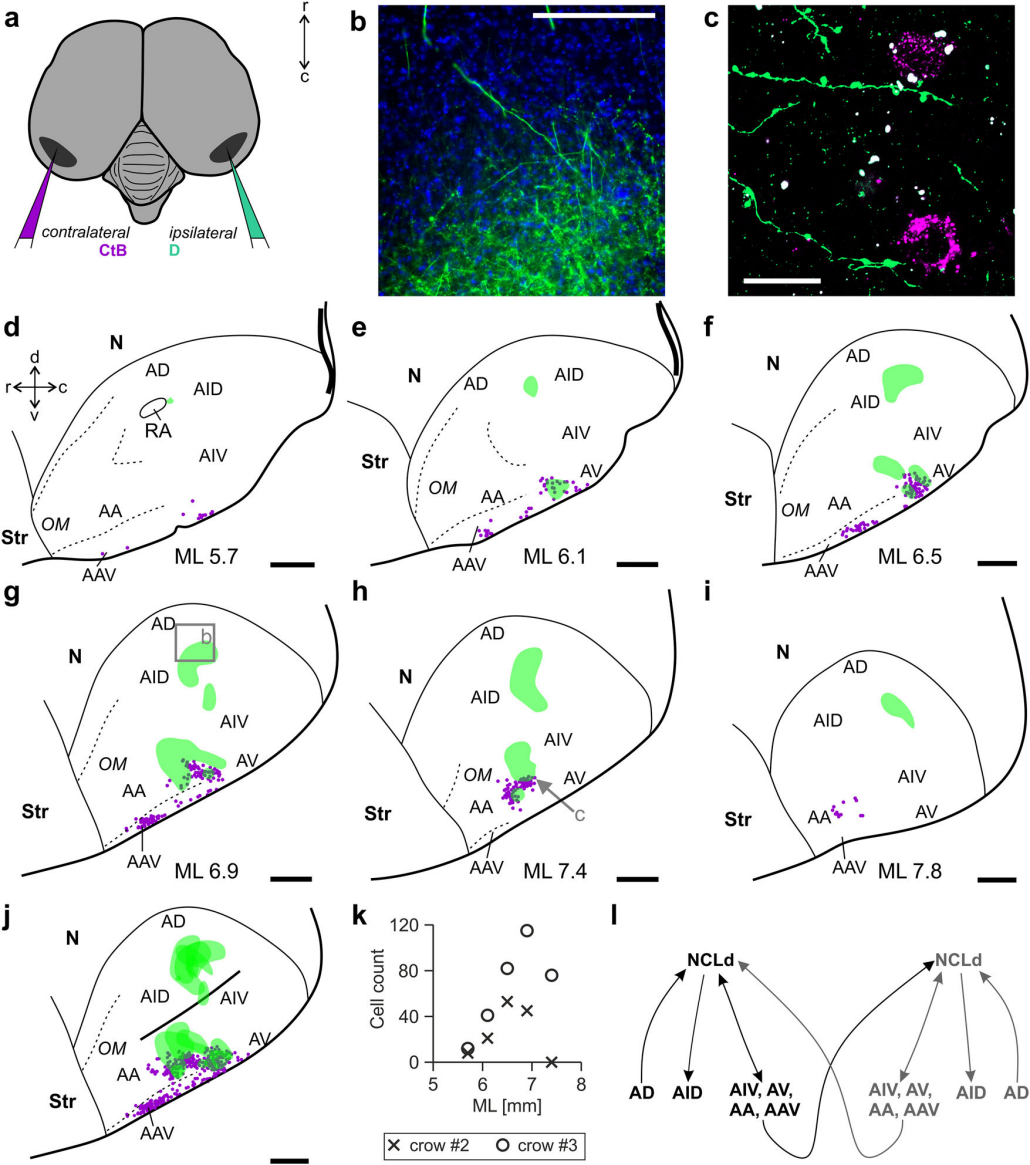
Colocalization of ipsilateral NCLd axons and arcopallial neurons projecting to the contralateral NCLd. ***a***, In the same bird, we injected an anterograde tracer (dextran) into the ipsilateral and a retrograde tracer (CTB) into the contralateral hemisphere. ***b***, Fluorophore labeled NCLd axons (green) terminating in the ipsilateral, dorsal intermediate arcopallium (AID). Note that the field of view corresponds to the inset shown in (***g***). DAPI stained nuclei are shown in blue. ***c***, Confocal image of ipsilateral NCLd axons (green) colocalized with two somata that are labeled with dye from the contralateral hemisphere (magenta). ***d–i***, Equally spaced sections of the arcopallium (sagittal view). Green shading indicates terminal fields of axons originating in the ipsilateral NCLd. Magenta dots mark individual cells projecting to the contralateral NCLd. ***j***, A projection image of all analyzed sections (***d–i***) reveals considerable overlap of NCLd axons (green) with cells projecting to the contralateral NCLd (magenta) in the ventral portion of the arcopallium (AA, AAV, AIV). ***k***, Numbers of arcopallial cells labeled by CTB injections into the contralateral NCLd for comparable slices from two crows (ML 5.7–7.4 in Figs. 6, 7; cf. Table 1). ***l***, Summary of all NCLd connections with the arcopallium described in the present study. Scale bars: ***b***, 200 µm; ***c***, 20 µm; ***d–j***, 1 mm, for abbreviations see Figure 6.

In previous reports, several distinct subregions have been identified within the songbird arcopallium (A), in both carrion crows and zebra finches (Mello et al., 2019; Kersten et al., 2022). We observed retrogradely labeled cells distributed along the mediolateral extent of the A (ML 2.0–ML 9.0; Fig. 4*b–i*), with the most medial cells located adjacent to the robust nucleus of the arcopallium (RA; Figs. 4*d*,*e*, 6*d*). At this mediolateral level, NCLd-projecting arcopallial cells were situated dorsal and ventral to RA (Fig. 6*d*,*e*). This dichotomic pattern was maintained toward lateral, with retrogradely labeled cells lining the dorsal and ventral borders of the A (Figs. 4*d–h*, 6*d–l*). Specifically, we observed a dorsal cluster of cells occupying the dorsal A (AD), with few cells extending into the dorsal intermediate A (AID) and a ventral cluster, which existed within aspects of the anterior, anterior ventral, medial ventral, and ventral A, with few cells extending into the ventral intermediate A (AA, AAV, AMV, AV, and AIV, respectively; Fig. 6*d–l*). Interestingly, only the ventral cluster of—ipsilaterally—NCLd-projecting cells was colocalized with cells that had taken up retrograde dye from an injection into the contralateral NCLd (Fig. 6*a–c*; *n* = 2 hemispheres in Crow 2; compare Table 1), consistent with the retrograde labeling observed in a second bird that received CTB only in the left hemisphere (Crow 3; Fig. 7*k*; compare Table 1). Most of the labeled ventral arcopallial cells projected either to the ipsi- or the contralateral NCLd (Fig. 6*d–l*; *n* = 2 hemispheres in Crow 2), highly reminiscent of an arcopallial cluster of cells projecting bilaterally to the dorsal NC in the zebra finch (Paterson and Bottjer, 2017). In the crow, few of these cells had taken up dye from both hemispheres (Fig. 6*c*,*e*,*i–k*; *n* = 2 hemispheres in Crow 2), which has not been reported in the zebra finch (Paterson and Bottjer, 2017).

The latter pool of contralaterally projecting, ventral arcopallial cells could potentially relay NCLd signals across hemispheres. Indeed, NCLd injections with an anterograde tracer produced prominent terminal fields within the ipsilateral arcopallium (Fig. 7*a–c*), which formed two distinct clusters (Fig. 7*j*; *n* = 3 hemispheres in 2 birds). In the ventral cluster, terminal fields overlapped with cells projecting to the contralateral NCLd and, thus, represent a potential disynaptic pathway connecting the NCLds of both hemispheres (Fig. 7*c–j*; *n* = 1; right hemisphere in Crow 2; compare Table 1). In the dorsal half of A, terminal fields were restricted to the AID (Fig. 7*b*,*d–j*; *n* = 3 hemispheres in 2 birds). This pathway has previously been described in the zebra finch (Wild and Farabaugh, 1996; Paterson and Bottjer, 2017) and appears to be the main premotor output of the NCLd (Zeier and Karten, 1971; Dubbeldam et al., 1997; Farries, 2001; Fernández et al., 2020b). Taken together, the interactions of the NCLd and the A seem rich and highly patterned, with input from AD, dense NCLd projections to the AID, and a cross-hemispheric, indirect connection of both NCLds via a field of ventral arcopallial cells (Fig. 7*l*).

### Subpallium

In contrast to other major subdivisions of the telencephalon, we did not identify any retrogradely labeled cells inside the striatum (Fig. 4*a–i*). However, we did observe a few NCLd-projecting cells in the ventral globus pallidus (GP; Fig. 4*e*), a sparse connection that has also been described in the pigeon (Kröner and Güntürkün, 1999). Conversely, anterograde labeling revealed many fibers in the medial-anterior aspect of the striatum, while we did not detect direct NCLd projections to the GP (data not shown; Fig. 10; Kersten et al., 2022).

### Diencephalon and midbrain

**Figure 8.**
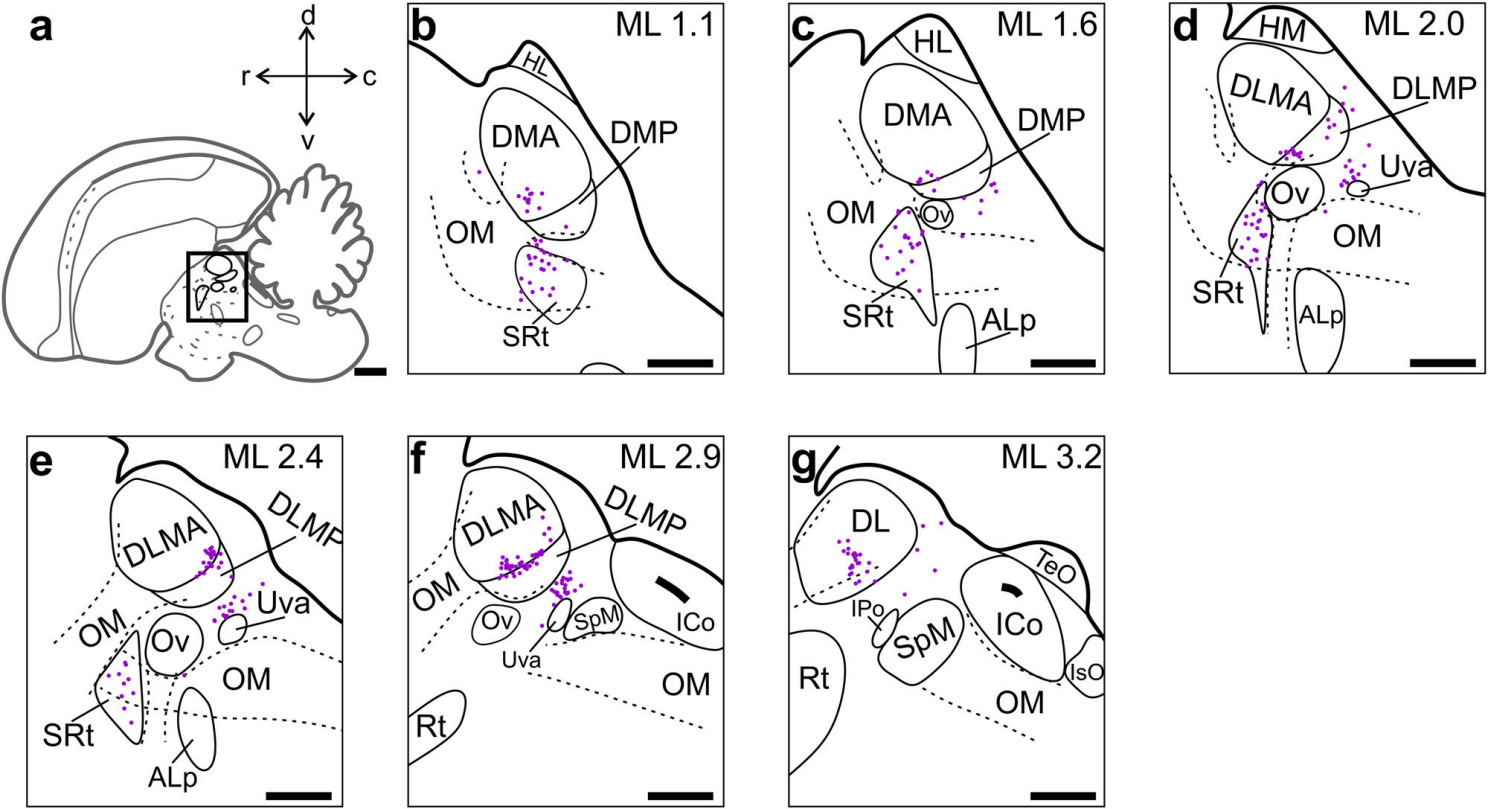
NCLd-projecting neurons in the diencephalon. ***a***, Schematic of the crow brain (sagittal view). The black box indicates the portion of the diencephalon in which we found retrogradely labeled somata (all projecting ipsilaterally). ***b–g***, Equally spaced sections of our region of interest within the diencephalon. Labeled somata were located in the dorsal nucleus of the thalamus, inside the SRt and in a distinct cluster adjacent to Uva. Scale bars: ***a***, 2 mm; ***b–g***, 1 mm. For abbreviations see Figure 4.

**Figure 9.**
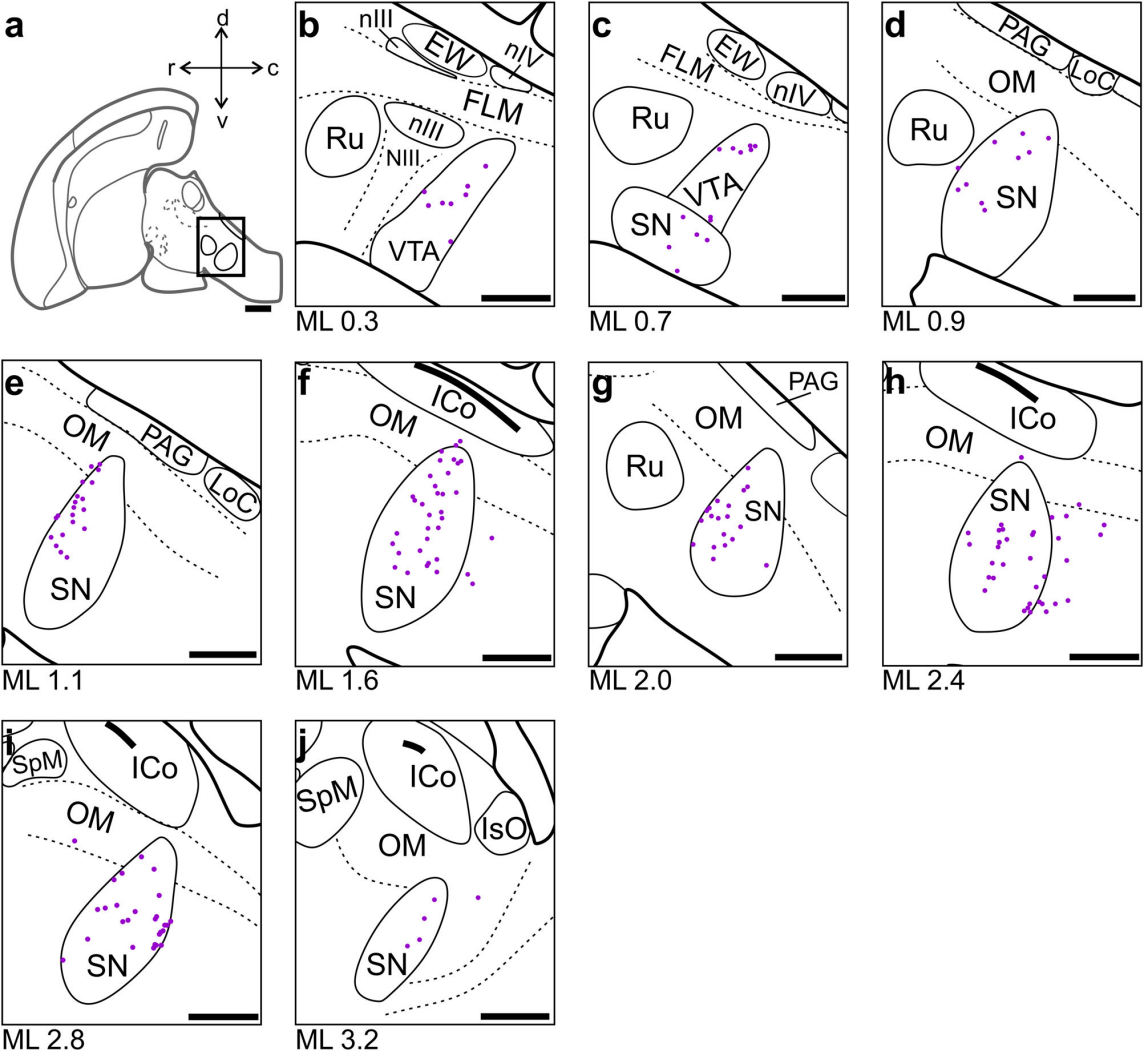
NCLd-projecting neurons in the midbrain. ***a***, Schematic of the crow brain (sagittal view). The black box indicates the portion of the mesencephalon in which we found retrogradely labeled somata (all projecting ipsilaterally). ***b–j***, Labeled somata were found throughout VTA and SN. Scale bars: ***a***, 2 mm; ***b–j***, 1 mm. For abbreviations see Figure 4.

We found that extratelencephalic afferents to NCLd were restricted to few defined areas in the thalamus and midbrain (Figs. 8*a*, 9*a*, respectively). In the thalamus, labeled cells were observed in all subregions of the dorsal thalamus (DMA, DMP, DLMA, DLMAP, and DL), inside the nucleus subrotundus (SRt), and in a narrow cluster just caudodorsal to the nucleus uvaeformis (Uva; Fig. 8*b–g*).

In the midbrain, we found robust labeling throughout the mediolateral extent of the ventral tegmental area (VTA; Fig. 9*b*,*c*) and the substantia nigra (SN; Fig. 9*c–j*). Importantly, these dopaminergic projections to NCLd have historically been used to define the extent of the nucleus (Divac and Mogensen, 1985; von Eugen et al., 2020).

### Overview

**Figure 10.**
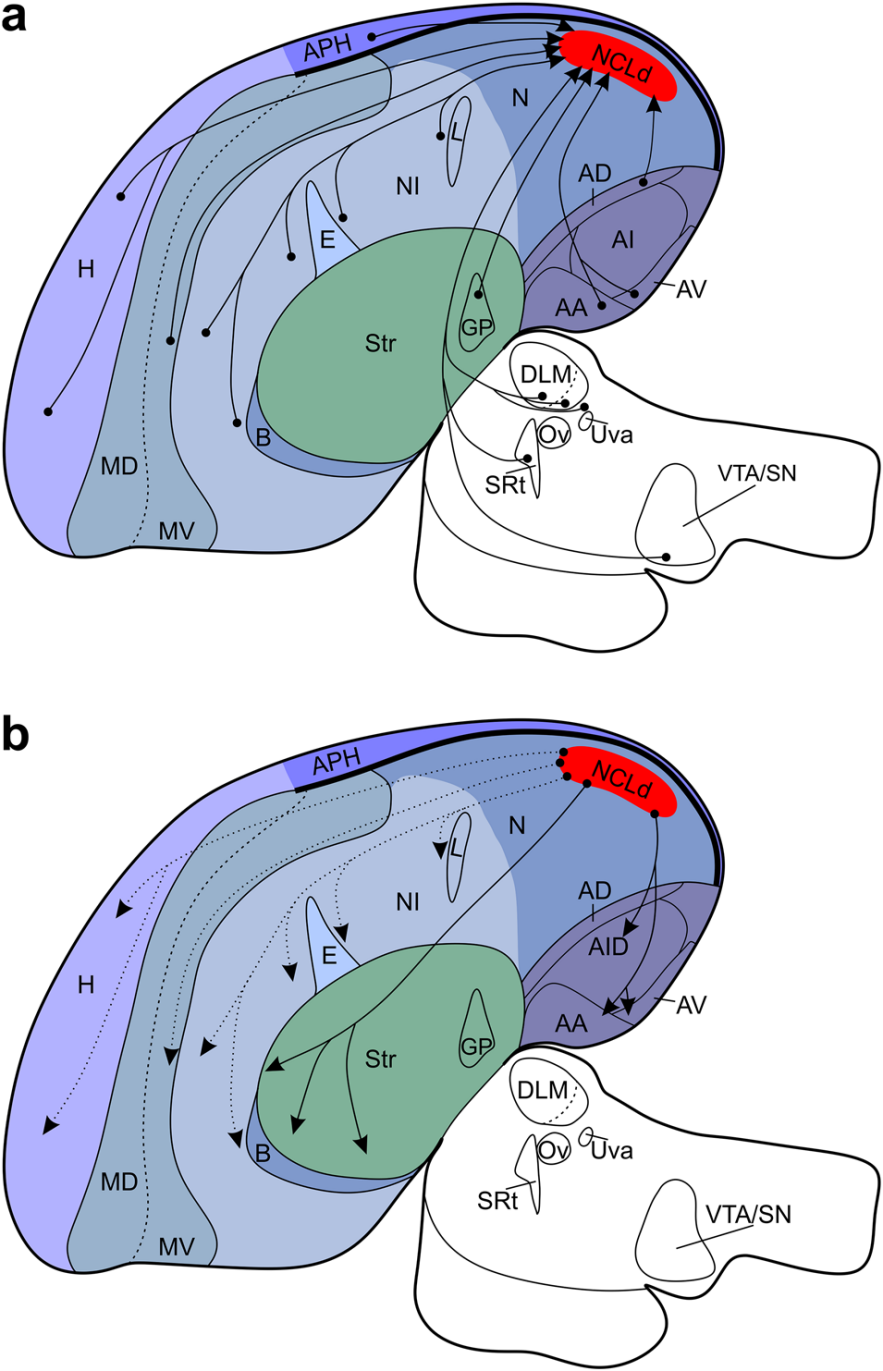
Connectivity diagram of the carrion crow's NCLd. ***a***, The NCLd receives input from secondary but not primary sensory areas of all modalities. Unidirectional NCLd inputs are received from the midbrain, thalamus, globus pallidus (GP), and hippocampal formation (HF), whereas the remaining depicted, afferent connections are likely reciprocal as shown in ***b***. ***b***, Strong, virtually unidirectional NCLd projections form dense terminal fields in the avian functional analog of the motor cortex, that is, the dorsal intermediate arcopallium (AID). Further dense, bidirectional connections overlap with ventral subdivisions of the arcopallium. Another unidirectional NCLd output targets the striatum. The remaining depicted projections were rarely observed in dense terminal fields. Given their presently more uncertain status, which can be confirmed by retrograde injections into individual target areas in the future, these connections are indicated with dashed lines. For abbreviations see Figure 4.

We registered the positions of individual afferent cells and the extent of distinct, efferent terminal fields to our atlas of the carrion crow brain (Kersten et al., 2022). Our results support the hypothesis that the monosynaptic connections of the carrion crow NCLd by and large resemble those previously described in the pigeon (Fig. 10; Kröner and Güntürkün, 1999; Fernández et al., 2020b). However, we found significant extensions compared with the pigeon data by reporting (1) input from the anterior half of the hippocampal formation to the NCLd, (2) arcopallial neurons that bilaterally project to the NCLd, and (3) a potentially songbird-specific NCLd-projecting cluster of cells adjacent to the thalamic song system nucleus Uva.

## Discussion

We have demonstrated the multimodal nature of the NCLd in a songbird species. In combination with previous reports on the pigeon NCL, our work suggests that the NCLd is a highly integrative hub, across avian taxa. Furthermore, our data add to a diverse set of studies that have argued for a critical premotor function of the NCLd via the AID (Yuan and Bottjer, 2020; Rinnert and Nieder, 2021; Kirschhock and Nieder, 2022; Zemel et al., 2023). The most surprising finding of our study was a dense cluster of NCLd-projecting cells at the rostral tip of the hippocampal formation. In the light of recent functional findings, these cells could provide highly processed spatial information, adding new evidence to the notion that the NCLd is an avian functional analog of the mammalian PFC (Payne et al., 2021; Sauer et al., 2022; Agarwal et al., 2023).

### New afferents from the hippocampal formation could provide spatial information to NCLd

Although the avian NCL is seen as the functional counterpart of the mammalian prefrontal cortex (PFC; Nieder, 2017), it is important to note that, in contrast to the PFC, no direct connection between the hippocampus and the NCL has been found (Leutgeb et al., 1996; Kröner and Güntürkün, 1999; Atoji et al., 2002; Atoji and Wild, 2006; Applegate et al., 2023a,b). Our findings further support the notion that the NCL does not send direct projections to the hippocampus. However, we found a previously undescribed, dense cluster of retrogradely labeled cells in the anterior portion of the hippocampal formation (Kersten et al., 2022). No such labeling has been reported in several previous studies that, like us, have injected CTB into the NCL in pigeons and zebra finches (Leutgeb et al., 1996; Kröner and Güntürkün, 1999; Paterson and Bottjer, 2017). These pigeon studies have reported NCL afferents from the medio-caudal hyperpallium, which, in turn, receives hippocampal input and is located just anterior to the hippocampal formation (Leutgeb et al., 1996; Kröner and Güntürkün, 1999; Atoji et al., 2002). This polysynaptic hippocampus→NCL connection in pigeons may be a mediator of the slow gamma-band coupling that has been observed between the two brain areas in pigeons during goal directed behavior (Zhao et al., 2019). Interestingly, our result suggests that the crow NCLd receives direct input from precisely the subdivision of the hippocampal formation that has recently been identified as the predominant location of “place cells” in songbirds and owls (Payne et al., 2021; Agarwal et al., 2023; Applegate et al., 2023b). Thus, spatial information relayed from these cells could guide the execution of corvid food caching and other spatial behaviors via the NCLd (Feenders et al., 2008; Bugnyar et al., 2016; Rinnert and Nieder, 2021; Applegate and Aronov, 2022).

In this context, we need to address the concern that the hippocampal labeling might have been caused by tracer spill-over into the dorsolateral corticoid area (CDL) that overlays the NCLd and contains fibers originating in the hippocampus (Atoji et al., 2002). At our injection coordinates, the CDL is a very thin layer of tissue that contains mostly fibers of passage and virtually no cell bodies (own observation and Applegate et al., 2023a). These fibers could, theoretically, have taken up tracer along our injection needle tract even though this phenomenon is thought to be minimal when dye coupled CTB is used (Chen and Aston-Jones, 1995; Conte et al., 2009). However, due to the anatomical arrangement of hippocampal projections within the CDL, we would—in the latter scenario—have expected labeled cells in the posterior part of the hippocampal formation but did not identify any (Atoji et al., 2002). Therefore, the intense hippocampal labeling reported by us suggests a previously undescribed projection from the hippocampal formation to the NCL.

### NCLd receives secondary visual and somatosensory afferents from the hyperpallium

The hyperpallium (H) or Wulst is composed of four layers in pigeons (Atoji and Wild, 2019). However, only the most ventral layer, the densocellular part of the hyperpallium (HD), and the dorsal part of the hyperpallium (HA) are readily identified in the crow and other songbirds (Reiner et al., 2004a; Jarvis et al., 2013; Lovell et al., 2020; Kersten et al., 2022). The HD has been renamed to MD and the HA is referred to as H in songbirds, based on cell clustering patterns (Jarvis et al., 2013; Kersten et al., 2022). MD and its overlaying thin layer, the intercalated hyperpallium (IH), contain primary sensory cells that receive visual or somatosensory information directly from dorsal aspects of the thalamus (Shimizu et al., 1995; Jarvis et al., 2013; Atoji and Wild, 2019; Stacho et al., 2020). This information is then relayed to the overlaying H, which consists of a rostral somatosensory and caudal visual subdivision (Stacho et al., 2020). Consequently, the hyperpallial labeling we found indicates that the NCLd receives secondary sensory information from both the somatosensory and the visual parts of the hyperpallium (Shimizu et al., 1995; Kröner and Güntürkün, 1999). In contrast, labeling was conspicuously absent in the thalamorecipient MD and IH, similar to reports that indicate that the PFC in mammals is not connected to thalamorecipient, primary sensory telencephalic areas (Karten et al., 1973; Miller and Cohen, 2001; Atoji and Wild, 2009, 2012; Jarvis et al., 2013). Therefore, the projection pattern in H is similar to N, which also relays secondary but not primary sensory information to the NCLd (see below; Kröner and Güntürkün, 1999; Stacho et al., 2020).

### Mesopallial and nidopallial NCLd afferents provide multimodal sensory information

Pioneering studies have reported sparsely distributed NCL-projecting cells inside the pigeon mesopallium (Leutgeb et al., 1996; Kröner and Güntürkün, 1999). The majority of these cells lined the border of MV and MD (previously termed M and HD, respectively; Reiner et al., 2004b; Jarvis et al., 2013). In the present study, most NCLd-projecting mesopallial cells were densely clustered around the crow MV's center of mass, an area likely equivalent to large aspects of the pigeon mesopallium intermedioventrale (MIV), which processes secondary sensory, tectofugal visual, and multisensory information (Atoji and Wild, 2012). Therefore, it stands to reason that the crow MV relays mostly visual and multisensory information to the NCLd areas covered by our injections (Atoji and Wild, 2012; Jarvis et al., 2013; Stacho et al., 2020).

In addition to the dense labeling around the central MV, our injections produced fewer labeled cells in the rostrolateral and caudal MV. Based on the connectivity of these areas in the pigeon and zebra finch, we speculate that these cells could relay secondary somatosensory and auditory information to the crow NCLd, respectively (Vates et al., 1996; Atoji and Wild, 2012; Ikeda et al., 2020). This is consistent with functional studies that have shown that crow NCLd neurons recorded from our injection area can be readily activated by auditory cues (Veit and Nieder, 2013; Moll and Nieder, 2015).

In the caudal nidopallium, our injections produced rich labeling within the borders of the crow NCLd and its periphery (von Eugen et al., 2020; Kersten et al., 2022). This finding demonstrates widespread local connectivity and indicates that the immunohistochemically identified body of NCLd is indeed a functional unit (Kröner and Güntürkün, 1999; Nieder, 2017; Fernández et al., 2020b). In contrast, the absence of NCLd connections to the NCLm and sparsely distributed CTB labeled cells in the ventral NC, which likely overlapped with the extent of NCLv, suggest limited direct interactions with these regions (Bottjer et al., 2000; von Eugen et al., 2020). However, more detailed investigations which are beyond the scope of the present study are needed to further resolve the local connectivity of identified NC subregions.

A comparison between the relative extent of the NCLd in carrion crows and zebra finches has demonstrated discernible interspecies differences (von Eugen et al., 2020). Therefore, it was unclear if the dorsal NCL connections that have been described in the zebra finch would also be revealed by our injections (Bottjer et al., 2000; Paterson and Bottjer, 2017; Bloomston et al., 2022). One major afferent connection of the zebra finch's dorsal NCL stems from the intermediate nidopallial area lMANshell (Bottjer et al., 2000; Paterson and Bottjer, 2017). We too found this NI connection to be one of the most prominent NCLd afferents inside the crow telencephalon and found additional cross-species similarities in the NCLd connections to the arcopallium (see below; Bottjer et al., 2000; Paterson and Bottjer, 2017). Therefore, the anatomical findings related to the zebra finch dorsal NCL could be largely applicable to the carrion crow (Bottjer et al., 2000; Paterson and Bottjer, 2017; Bloomston et al., 2022).

Our findings confirm that NI, like MV, is a major source of multimodal secondary but not primary sensory input and is reciprocally connected to the NCLd (Leutgeb et al., 1996; Kröner and Güntürkün, 1999; Fernández et al., 2020b). NI and MV are interconnected regions that are both involved in visual processing via the tectofugal pathway and have been jointly described under the umbrella term “medial nidopallium/mesopallium” (Atoji and Wild, 2012; Behroozi et al., 2020; Hahn and Rose, 2023). In pigeons, the NI part of this cluster is involved in the execution of targeted pecking (Helduser and Güntürkün, 2012; Helduser et al., 2013; Hahn and Rose, 2023). Our data provide further evidence for the hypothesis that this likely happens in concert with neurons in the NCL (Hahn and Rose, 2023), which can exhibit a motor preparatory signal related to the number or position of targeted pecks in the carrion crow (Rinnert and Nieder, 2021; Kirschhock and Nieder, 2022). Taken together, the combined inputs from NI and MV provide the NCLd with preprocessed, multimodal sensory information that can be integrated by abundant local connections, to give rise to higher cognitive functions (Güntürkün and Bugnyar, 2016; Nieder, 2017; Nieder et al., 2020).

### The NCLd is connected to functionally distinct arcopallial regions

NCLd connections to the arcopallium were roughly split into a dorsal and a ventral cluster, consistent with previous studies characterizing the projections of the caudal nidopallium to the arcopallium (Paterson and Bottjer, 2017; Bloomston et al., 2022). In its dorsal half, the arcopallium is composed of two overlying fields, the AID and the AD (Mello et al., 2019; Kersten et al., 2022). The AID projects to the basal ganglia, motor thalamus, tectum, and reticular formation (Bottjer et al., 2000; Fernández et al., 2020b), and functional studies suggest that the AID has a critical role in various motor behaviors, in particular movements of the head and neck (Levine and Zeigler, 1981; Knudsen et al., 1995; Wild and Krützfeldt, 2012; Mandelblat-Cerf et al., 2014). Therefore, our finding of dense terminal fields within the AID supports the notion that the NCLd serves as a key executive premotor structure (Zeier and Karten, 1971; Dubbeldam et al., 1997; Farries, 2001; Feenders et al., 2008; Yuan and Bottjer, 2020; Fernández et al., 2020a). In contrast to the AID, not much is known about the function of AD from which the NCLd received the bulk of its dorsal arcopallial input. Interestingly, the extent of our dorsal field of retrogradely labeled cells matched well with the distribution of the genetic marker CBLN2, which selectively indicates the AD in the zebra finch (Mello et al., 2019). Several lines of evidence indicate that the AD and AID receive markedly different neuromodulatory inputs suggesting that the NCLd recipient AID population and the NCLd-projecting AD population may serve different functions (Mello et al., 2019; Sen et al., 2019).

In the ventral half of the arcopallium (i.e., AA, AAV, AMV, AV, and AIV combined), cells labeled by ipsilateral NCLd injections were colocalized with cells labeled by contralateral injections. Among these NCLd afferents, which have been described previously, we found few double labeled cells projecting to the ipsi- and contralateral NCLd that have not been reported yet (Paterson and Bottjer, 2017). The lack of such cells in the Paterson and Bottjer (2017) study may be explained by offset NCL injections between hemispheres, if the arcopallium→NCL projections are topographic. Similarly, the low number of cells we found may be an underestimate and a consequence of within NCLd injection site variability. In the zebra finch, Paterson and Bottjer (2017) found lMANshell neurons that projected to both the ventral arcopallium and the dorsal NCL (“NCLshell”). They concluded that the ventral arcopallium is a point of convergence of information from the anterior and the caudal nidopallium, which is then sent to the contralateral hemisphere (Paterson and Bottjer, 2017). Furthermore, a subset of our contralaterally projecting ventral arcopallial neurons was colocalized with dense terminal fields originating in the ipsilateral NCLd. Therefore, we suggest that the ventral portion of the arcopallium could be a critical node within a loop that includes the NCLd and the NI (i.e., lMANshell region) within and across hemispheres (Johnson et al., 1995; Paterson and Bottjer, 2017).

In the medioventral part of the arcopallium, NCLd-projecting cells overlapped with the AMV, a structure that was previously also referred to as nucleus taenia (Mello et al., 2019). This nucleus has been reported to influence affective states via its projections to the hypothalamus (Zeier and Karten, 1971; Thompson et al., 1998; Cheng et al., 1999).

### Few, defined dorsal thalamic areas project to the NCLd

In mammals, the initiation of cued movements and the maintenance of working memory depends on thalamic input to the prefrontal and motor cortices (Guo et al., 2017; Gaidica et al., 2018; Sauerbrei et al., 2020; Dacre et al., 2021; Inagaki et al., 2022). Similar to the PFC, the crow NCLd is involved in working memory tasks and the execution of cued movements and these functions could depend on its connections to the thalamus as well (Veit et al., 2014; Rinnert and Nieder, 2021; Kirschhock and Nieder, 2022). Indeed, as in the pigeon, we found NCLd-projecting thalamic cells in the dorsal thalamus and SRt (Leutgeb et al., 1996; Kröner and Güntürkün, 1999). Additionally, we discovered a previously undescribed distinct cluster of labeled cells just dorsocaudal to the songbird specific Uva. Together, these nuclei relay multisensory and cerebellar information to the striatum and pallium (Miceli and Repérant, 1985; Veenman et al., 1995; Wild and Gaede, 2016; Nicholson et al., 2018). Therefore, they could provide short latency, movement-triggering signals to the NCLd, paralleling a recently described functional thalamonidopallial connection in the song system (Inagaki et al., 2022; Moll et al., 2023).

### Dopaminergic input to the NCLd could facilitate behavioral flexibility

The extent of NCL is typically defined by its dense dopaminergic innervation (Divac and Mogensen, 1985; von Eugen et al., 2020). These dopaminergic fibers originate in the SN and VTA, consistent with the robust CTB labeling we found in those nuclei (Kröner and Güntürkün, 1999). Interestingly, parallel dopaminergic signals from the midbrain's periaqueductal gray (PAG) to the dorsal nidopallium (i.e., HVC) are critical for rapid sensory-motor learning in vocalizing zebra finches (Tanaka et al., 2018). It is thus tempting to speculate that the carrion crow's highly flexible learning abilities could be supported by analog mechanisms (Nieder, 2017; Ott and Nieder, 2019).

## Reporting Summary

Further information on research design is available in the Nature Research Reporting Summary linked to this article.

## Data Availability

The datasets generated during and/or analyzed during the current study are available from the corresponding author on reasonable request. The data that support the findings of this study are available from the corresponding author upon reasonable request.

## Synthesis

Reviewing Editor: David Schoppik, New York University Decisions are customarily a result of the Reviewing Editor and the peer reviewers coming together and discussing their recommendations until a consensus is reached. When revisions are invited, a fact-based synthesis statement explaining their decision and outlining what is needed to prepare a revision will be listed below. The following reviewer(s) agreed to reveal their identity: NONE. Note: If this manuscript was transferred from JNeurosci and a decision was made to accept the manuscript without peer review, a brief statement to this effect will instead be what is listed below.

The revised manuscript addresses the reviewers' concerns.

## References

[B1] Agarwal A, Sarel A, Dori D, Ulanovsky N, Gutfreund Y (2023) Spatial coding in the hippocampus and hyperpallium of flying owls. Proc Natl Acad Sci U S A 120:e2212418120. 10.1073/pnas.2212418120 36693104 PMC9945993

[B2] Applegate MC, Aronov D (2022) Flexible use of memory by food-caching birds. Elife 11:e70600. 10.7554/eLife.70600 35467526 PMC9038193

[B3] Applegate MC, Gutnichenko KS, Aronov D (2023a) Topography of inputs into the hippocampal formation of a food-caching bird. bioRxiv.

[B4] Applegate MC, Gutnichenko KS, Mackevicius EL, Aronov D (2023b) An entorhinal-like region in food-caching birds. Curr Biol 33:2465–2477. 10.1016/j.cub.2023.05.031 37295426 PMC10329498

[B5] Atoji Y, Wild JM (2006) Anatomy of the avian hippocampal formation. Rev Neurosci 17:3–16. 10.1515/REVNEURO.2006.17.1-2.3 16703939

[B6] Atoji Y, Wild JM (2009) Afferent and efferent projections of the central caudal nidopallium in the pigeon (*Columba livia*). J Comp Neurol 517:350–370. 10.1002/cne.22146 19760740

[B7] Atoji Y, Wild JM (2012) Afferent and efferent projections of the mesopallium in the pigeon (*Columba livia*). J Comp Neurol 520:717–741. 10.1002/cne.22763 21935938

[B8] Atoji Y, Wild JM (2019) Projections of the densocellular part of the hyperpallium in the rostral Wulst of pigeons (*Columba livia*). Brain Res 1711:130–139. 10.1016/j.brainres.2019.01.001 30610876

[B9] Atoji Y, Wild JM, Yamamoto Y, Suzuki Y (2002) Intratelencephalic connections of the hippocampus in pigeons (*Columba livia*). J Comp Neurol 447:177–199. 10.1002/cne.10239 11977120

[B10] Behroozi M, et al. (2020) Event-related functional MRI of awake behaving pigeons at 7T. Nat Commun 11:4715. 10.1038/s41467-020-18437-1 32948772 PMC7501281

[B11] Bird CD, Emery NJ (2009) Insightful problem solving and creative tool modification by captive nontool-using rooks. Proc Natl Acad Sci U S A 106:10370–10375. 10.1073/pnas.0901008106 19478068 PMC2700937

[B12] Bloomston NA, Zaharas K, Lawley K, Fenn T, Person E, Huber H, Zhang Z, Prather JF (2022) Exploring links from sensory perception to movement and behavioral motivation in the caudal nidopallium of female songbirds. J Comp Neurol 530:1622–1633. 10.1002/cne.25305 35073426 PMC9119909

[B13] Bottjer SW, Brady JD, Cribbs B (2000) Connections of a motor cortical region in zebra finches: relation to pathways for vocal learning. J Comp Neurol 420:244–260. 10.1002/(SICI)1096-9861(20000501)420:2<244::AID-CNE7>3.0.CO;2-M 10753310

[B14] Bugnyar T, Reber SA, Buckner C (2016) Ravens attribute visual access to unseen competitors. Nat Commun 7:3–8. 10.1038/ncomms10506 PMC4740864 26835849

[B15] Chen S, Aston-Jones G (1995) Evidence that cholera toxin B subunit (CTb) can be avidly taken up and transported by fibers of passage. Brain Res 674:107–111. 10.1016/0006-8993(95)00020-Q 7773677

[B16] Cheng MF, Chaiken M, Zuo M, Miller H (1999) Nucleus taenia of the amygdala of birds: anatomical and functional studies in ring doves (*Streptopelia risoria*) and European starlings (*Sturnus vulgaris*). Brain Behav Evol 53:243–270. 10.1159/000006597 10473902

[B17] Conte WL, Kamishina H, Reep RL (2009) Multiple neuroanatomical tract-tracing using fluorescent Alexa Fluor conjugates of cholera toxin subunit B in rats. Nat Protoc 4:1157–1166. 10.1038/nprot.2009.93 19617887

[B18] Dacre J, et al. (2021) A cerebellar-thalamocortical pathway drives behavioral context-dependent movement initiation. Neuron 109:2326–2338. 10.1016/j.neuron.2021.05.016 34146469 PMC8315304

[B19] Ditz HM, Nieder A (2015) Neurons selective to the number of visual items in the corvid songbird endbrain. Proc Natl Acad Sci U S A 112:7827–7832. 10.1073/pnas.1504245112 26056278 PMC4485087

[B20] Ditz HM, Nieder A (2016) Sensory and working memory representations of small and large numerosities in the crow endbrain. J Neurosci 36:12044–12052. 10.1523/JNEUROSCI.1521-16.2016 27881787 PMC6604915

[B21] Divac I, Mogensen J (1985) The prefrontal “cortex” in the pigeon catecholamine histofluorescence. Neuroscience 15:677–682. 10.1016/0306-4522(85)90069-7 4069352

[B22] Dubbeldam JL, Den Boer-Visser AM, Bout RG (1997) Organization and efferent connections of the archistriatum of the mallard, *Anas platyrhynchos* L.: an anterograde and retrograde tracing study. J Comp Neurol 388:632–657. 10.1002/(SICI)1096-9861(19971201)388:4<632::AID-CNE10>3.0.CO;2-N 9388021

[B23] Düring DN, et al. (2020) Fast retrograde access to projection neuron circuits underlying vocal learning in songbirds. Cell Rep 33:1–11. 10.1016/j.celrep.2020.108364 PMC8236207 33176132

[B24] Farries MA (2001) The oscine song system considered in the context of the avian brain: lessons learned from comparative neurobiology. Brain Behav Evol 58:80–100. 10.1159/000047263 11805375

[B25] Feenders G, Liedvogel M, Rivas M, Zapka M, Horita H, Hara E, Wada K, Mouritsen H, Jarvis ED (2008) Molecular mapping of movement-associated areas in the avian brain: a motor theory for vocal learning origin. PLoS One 3:1–27. 10.1371/journal.pone.0001768 PMC2258151 18335043

[B26] Fernández M, Ahumada-Galleguillos P, Sentis E, Marín G, Mpodozis J (2020a) Intratelencephalic projections of the avian visual dorsal ventricular ridge: laminarly segregated, reciprocally and topographically organized. J Comp Neurol 528:321–359. 10.1002/cne.24757 31407337

[B27] Fernández M, Morales C, Durán E, Fernández-Colleman S, Sentis E, Mpodozis J, Karten HJ, Marín GJ (2020b) Parallel organization of the avian sensorimotor arcopallium: tectofugal visual pathway in the pigeon (*Columba livia*). J Comp Neurol 528:597–623. 10.1002/cne.24775 31531866

[B28] Gagliardo A, Bonadonna F, Divac I (1996) Behavioural effects of ablations of the presumed “prefrontal cortex” or the corticoid in pigeons. Behav Brain Res 78:155–162. 10.1016/0166-4328(95)00243-X 8864047

[B29] Gagliardo A, Mazzotto M, Divac I (1997) Memory of radial maze behavior in pigeons after ablations of the presumed equivalent of mammalian prefrontal cortex. Behav Neurosci 111:955–962. 10.1037/0735-7044.111.5.968 9383516

[B30] Gaidica M, Hurst A, Cyr C, Leventhal DK (2018) Distinct populations of motor thalamic neurons encode action initiation, action selection, and movement vigor. J Neurosci 38:6563–6573. 10.1523/JNEUROSCI.0463-18.2018 29934350 PMC6052244

[B31] Güntürkün O (1997) Cognitive impairments after lesions of the neostriatum caudolaterale and its thalamic afferent in pigeons: functional similarities to the mammalian prefrontal system? J Hirnforsch 38:133–143. PMID: 9059925

[B32] Güntürkün O (2005) The avian ‘prefrontal cortex’ and cognition. Curr Opin Neurobiol 15:686–693. 10.1016/j.conb.2005.10.003 16263260

[B33] Güntürkün O, Bugnyar T (2016) Cognition without cortex. Trends Cogn Sci 20:291–303. 10.1016/j.tics.2016.02.001 26944218

[B34] Guo ZV, Inagaki HK, Daie K, Druckmann S, Gerfen CR, Svoboda K (2017) Maintenance of persistent activity in a frontal thalamocortical loop. Nature 545:181–186. 10.1038/nature22324 28467817 PMC6431254

[B35] Hahn LA, Rose J (2023) Executive control of sequence behavior in pigeons involves two distinct brain regions. eNeuro 10:1–11. 10.1523/ENEURO.0296-22.2023 PMC9997693 36849259

[B36] Hartmann B, Güntürkün O (1998) Selective deficits in reversal learning after neostriatum caudolaterale lesions in pigeons: possible behavioral equivalencies to the mammalian prefrontal system. Behav Brain Res 96:125–133. 10.1016/S0166-4328(98)00006-0 9821549

[B37] Helduser S, Cheng S, Güntürkün O (2013) Identification of two forebrain structures that mediate execution of memorized sequences in the pigeon. J Neurophysiol 109:958–968. 10.1152/jn.00763.2012 23236000

[B38] Helduser S, Güntürkün O (2012) Neural substrates for serial reaction time tasks in pigeons. Behav Brain Res 230:132–143. 10.1016/j.bbr.2012.02.013 22348895

[B39] Herculano-Houzel S (2009) The human brain in numbers: a linearly scaled-up primate brain. Front Hum Neurosci 3:31. 10.3389/neuro.09.031.2009 19915731 PMC2776484

[B40] Herculano-Houzel S (2011) Brains matter, bodies maybe not: the case for examining neuron numbers irrespective of body size. Ann N Y Acad Sci 1225:191–199. 10.1111/j.1749-6632.2011.05976.x 21535005

[B41] Hoffmann A, Rüttler V, Nieder A (2011) Ontogeny of object permanence and object tracking in the carrion crow, *Corvus corone*. Anim Behav 82:359–367. 10.1016/j.anbehav.2011.05.012

[B42] Hunt GR (1996) Manufacture and use of hook-tools by new Caledonian crows. Nature 379:249–251. 10.1038/379249a0

[B43] Ikeda MZ, Trusel M, Roberts TF (2020) Memory circuits for vocal imitation. Curr Opin Neurobiol 60:37–46. 10.1016/j.conb.2019.11.002 31810009 PMC7694441

[B44] Inagaki HK, et al. (2022) A midbrain-thalamus-cortex circuit reorganizes cortical dynamics to initiate movement. Cell 185:1065–1081. 10.1016/j.cell.2022.02.006 35245431 PMC8990337

[B45] Iwaniuk AN, Hurd PL (2005) The evolution of cerebrotypes in birds. Brain Behav Evol 65:215–230. 10.1159/000084313 15761215

[B46] Jarvis E, et al. (2005) Avian brains and a new understanding of vertebrate brain evolution. Nat Rev Neurosci 6:151–159. 10.1038/nrn1606 15685220 PMC2507884

[B47] Jarvis ED, et al. (2013) Global view of the functional molecular organization of the avian cerebrum: mirror images and functional columns. J Comp Neurol 521:3614–3665. 10.1002/cne.23404 23818122 PMC4145244

[B48] Johnson F, Sablan MM, Bottjer SW (1995) Topographic organization of a forebrain pathway involved with vocal learning in zebra finches. J Comp Neurol 358:260–278. 10.1002/cne.903580208 7560286

[B49] Kabadayi C, Osvath M (2017) Ravens parallel great apes in flexible planning for tool-use and bartering. Science 357:202–204. 10.1126/science.aam8138 28706072

[B50] Karten HJ (2015) Vertebrate brains and evolutionary connectomics: on the origins of the mammalian ‘neocortex.’. Philos Trans R Soc Lond B Biol Sci 370:1–12. 10.1098/rstb.2015.0060 PMC4650131 26554047

[B51] Karten HJ, Hodos W, Nauta WJH, Revzin AM (1973) Neural connections of the “visual wulst” of the avian telencephalon. Experimental studies in the pigeon (*Columba livia*) and owl (*Speotyto cunicularia*). J Comp Neurol 150:253–277. 10.1002/cne.901500303 4721779

[B52] Kersten Y, Friedrich-Müller B, Nieder A (2021) A histological study of the song system of the carrion crow (*Corvus corone*). J Comp Neurol 529:2576–2595. 10.1002/cne.25112 33474740

[B53] Kersten Y, Friedrich-Müller B, Nieder A (2022) A brain atlas of the carrion crow (*Corvus corone*). J Comp Neurol 530:3011–3038. 10.1002/cne.25392 35938778

[B54] Kirschhock ME, Nieder A (2022) Number selective sensorimotor neurons in the crow translate perceived numerosity into number of actions. Nat Commun 13:6913. 10.1038/s41467-022-34457-5 36376297 PMC9663431

[B55] Kirschhock ME, Nieder A (2023) Association neurons in the crow telencephalon link visual signs to numerical values. Proc Natl Acad Sci U S A 120:e2313923120. 10.1073/pnas.2313923120 37903264 PMC10636302

[B56] Knudsen EI, Cohen YE, Masino T (1995) Characterization of a forebrain gaze field in the archistriatum of the barn owl: microstimulation and anatomical connections. J Neurosci 15:5139–5151. 10.1523/JNEUROSCI.15-07-05139.1995 7623141 PMC6577889

[B57] Kröner S, Güntürkün O (1999) Afferent and efferent connections of the caudolateral neostriatum in the pigeon (*Columba livia*): a retro- and anterograde pathway tracing study. J Comp Neurol 407:228–260. 10.1002/(SICI)1096-9861(19990503)407:2<228::AID-CNE6>3.0.CO;2-2 10213093

[B58] Kubke MF, Massoglia DP, Carr CE (2004) Bigger brains or bigger nuclei? Regulating the size of auditory structures in birds. Brain Behav Evol 63:169–180. 10.1159/000076242 14726625 PMC3269630

[B59] Kverková K, et al. (2022) The evolution of brain neuron numbers in amniotes. Proc Natl Acad Sci U S A 119:e2121624119. 10.1073/pnas.2121624119 35254911 PMC8931369

[B60] Leutgeb S, Husband S, Riters LV, Shimizu T, Bingman VP (1996) Telencephalic afferents to the caudolateral neostriatum of the pigeon. Brain Res 730:173–181. 10.1016/0006-8993(96)00444-1 8883901

[B61] Levine RR, Zeigler HP (1981) Extratelencephalic pathways and feeding behavior in the pigeon (*Columba livia*). Brain Behav Evol 19:74–92. 10.1159/000121635 7326570

[B62] Lovell PV, Wirthlin M, Kaser T, Buckner AA, Carleton JB, Snider BR, Mchugh AK, Tolpygo A, Mitra PP, Mello CV (2020) ZEBrA-zebra finch expression brain atlas: a resource for comparative molecular and neuroanatomy and brain evolution studies. J Comp Neurol 528:2099–2131. 10.1002/cne.24879 32037563 PMC8219259

[B63] Mandelblat-Cerf Y, Las L, Denisenko N, Fee MS (2014) A role for descending auditory cortical projections in songbird vocal learning. Elife 3:e02152. 10.7554/eLife.02152 24935934 PMC4113997

[B64] Mehlhorn J, Hunt GR, Gray RD, Rehkämper G, Güntürkün O (2010) Tool-making new caledonian crows have large associative brain areas. Brain Behav Evol 75:63–70. 10.1159/000295151 20215728

[B65] Mello CV, Clayton DF (1994) Song-induced ZENK gene expression in auditory pathways of songbird brain and its relation to the song control system. J Neurosci 14:6652–6666. 10.1523/JNEUROSCI.14-11-06652.1994 7965067 PMC6577290

[B66] Mello CV, Kaser T, Buckner AA, Wirthlin M, Lovell PV (2019) Molecular architecture of the zebra finch arcopallium. J Comp Neurol 527:2512–2556. 10.1002/cne.24688 30919954 PMC6879308

[B67] Mello CV, Vates GE, Okuhata S, Nottebohm F (1998) Descending auditory pathways in the adult male zebra finch (*Taeniopygia guttata*). J Comp Neurol 395:137–160. 10.1002/(SICI)1096-9861(19980601)395:2<137::AID-CNE1>3.0.CO;2-3 9603369

[B68] Miceli D, Repérant J (1985) Telencephalic afferent projections from the diencephalon and brainstem in the pigeon. A retrograde multiple-label fluorescent study. Exp Biol 44:71–99. PMID: 3850028

[B69] Miller EK, Cohen JD (2001) An integrative theory of prefrontal cortex function. Annu Rev Neurosci 24:167–202. 10.1146/annurev.neuro.24.1.167 11283309

[B70] Mogensen J, Divac I (1982) The prefrontal “cortex” in the pigeon: behavioral evidence. Brain Behav Evol 21:60–66. 10.1159/000121617 7159828

[B71] Mogensen J, Divac I (1993) Behavioural effects of ablation of the pigeon-equivalent of the mammalian prefrontal cortex. Behav Brain Res 55:101–107. 10.1016/0166-4328(93)90012-F 8329122

[B72] Moll FW, Kranz D, Corredera Asensio A, Elmaleh M, Ackert-Smith LA, Long MA (2023) Thalamus drives vocal onsets in the zebra finch courtship song. Nature 616:132–136. 10.1038/s41586-023-05818-x 36949189 PMC11967199

[B73] Moll FW, Nieder A (2014) The long and the short of it: rule-based relative length discrimination in carrion crows, *Corvus corone*. Behav Processes 107:142–149. 10.1016/j.beproc.2014.08.009 25151937

[B74] Moll FW, Nieder A (2015) Cross-modal associative mnemonic signals in crow endbrain neurons. Curr Biol 25:2196–2201. 10.1016/j.cub.2015.07.013 26255848

[B75] Moll FW, Nieder A (2017) Modality-invariant audio-visual association coding in crow endbrain neurons. Neurobiol Learn Mem 137:65–76. 10.1016/j.nlm.2016.11.011 27864088

[B76] Nicholson DA, Roberts TF, Sober SJ (2018) Thalamostriatal and cerebellothalamic pathways in a songbird, the Bengalese finch. J Comp Neurol 526:1550–1570. 10.1002/cne.24428 29520771 PMC5899675

[B77] Nieder A (2017) Inside the corvid brain—probing the physiology of cognition in crows. Curr Opin Behav Sci 16:8–14. 10.1016/j.cobeha.2017.02.005

[B78] Nieder A (2023) Neuroscience of cognitive control in crows. Trends Neurosci 46:783–785. 10.1016/j.tins.2023.07.002 37524636

[B79] Nieder A, Wagener L, Rinnert P (2020) A neural correlate of sensory consciousness in a corvid bird. Science 369:1626–1629. 10.1126/science.abb1447 32973028

[B80] Ott T, Nieder A (2019) Dopamine and cognitive control in prefrontal cortex. Trends Cogn Sci 23:213–234. 10.1016/j.tics.2018.12.006 30711326

[B81] Paterson AK, Bottjer SW (2017) Cortical inter-hemispheric circuits for multimodal vocal learning in songbirds. J Comp Neurol 525:3312–3340. 10.1002/cne.24280 28681379 PMC6301027

[B82] Payne HL, Lynch GF, Aronov D (2021) Neural representations of space in the hippocampus of a food-caching bird. Science 373:343–348. 10.1126/science.abg2009 34437154 PMC8503942

[B83] Rehkämper G, Frahm HD, Zilles K (1991) Quantitative development of brain and brain structures in birds (galliformes and passeriformes) compared to that in mammals (insectivores and primates) (part 1 of 2). Brain Behav Evol 37:125–134. 10.1159/000114353 2070254

[B84] Reiner A, et al. (2004a) Revised nomenclature for avian telencephalon and some related brainstem nuclei. J Comp Neurol 473:377–414. 10.1002/cne.20118 15116397 PMC2518311

[B85] Reiner A, Perkel DJ, Mello CV, Jarvis ED (2004b) Songbirds and the revised avian brain nomenclature. Ann N Y Acad Sci 1016:77–108. 10.1196/annals.1298.013 15313771 PMC2481519

[B86] Rinnert P, Kirschhock ME, Nieder A (2019) Neuronal correlates of spatial working memory in the endbrain of crows. Curr Biol 29:2616–2624.e4. 10.1016/j.cub.2019.06.060 31378607

[B87] Rinnert P, Nieder A (2021) Neural code of motor planning and execution during goal-directed movements in crows. J Neurosci 41:4060–4072. 10.1523/JNEUROSCI.0739-20.2021 33608384 PMC8176758

[B88] Rose J, Colombo M (2005) Neural correlates of executive control in the avian brain. PLoS Biol 3:e190. 10.1371/journal.pbio.0030190 15941358 PMC1088974

[B89] Sadananda M, Bischof HJ (2006) Afferentation of the lateral nidopallium: a tracing study of a brain area involved in sexual imprinting in the zebra finch (*Taeniopygia guttata*). Brain Res 1106:111–122. 10.1016/j.brainres.2006.04.009 16843442

[B90] Sauer JF, Folschweiller S, Bartos M (2022) Topographically organized representation of space and context in the medial prefrontal cortex. Proc Natl Acad Sci U S A 119:4–11. 10.1073/pnas.2117300119 PMC8833199 35121665

[B91] Sauerbrei BA, Guo J-Z, Cohen JD, Mischiati M, Guo W, Kabra M, Verma N, Mensh B, Branson K, Hantman AW (2020) Cortical pattern generation during dexterous movement is input-driven. Nature 577:386–391. 10.1038/s41586-019-1869-9 31875851 PMC6962553

[B92] Schindelin J, et al. (2012) Fiji: an open-source platform for biological-image analysis. Nat Methods 9:676–682. 10.1038/nmeth.2019 22743772 PMC3855844

[B93] Sen S, Parishar P, Pundir AS, Reiner A, Iyengar S (2019) The expression of tyrosine hydroxylase and DARPP-32 in the house crow (*Corvus splendens*) brain. J Comp Neurol 527:1801–1836. 10.1002/cne.24649 30697741

[B94] Shimizu T, Cox K, Karten HJ (1995) Intratelencephalic projections of the visual wulst in pigeons (*Columba livia*). J Comp Neurol 359:551–572. 10.1002/cne.903590404 7499547

[B95] Stacho M, Herold C, Rook N, Wagner H, Axer M, Amunts K, Güntürkün O (2020) A cortex-like canonical circuit in the avian forebrain. Science 369:1–12. 10.1126/science.abc5534 32973004

[B96] Striedter GF (2016) Evolution of the hippocampus in reptiles and birds. J Comp Neurol 524:496–517. 10.1002/cne.23803 25982694

[B97] Ströckens F, Neves K, Kirchem S, Schwab C, Herculano-Houzel S, Güntürkün O (2022) High associative neuron numbers could drive cognitive performance in corvid species. J Comp Neurol 530:1588–1605. 10.1002/cne.25298 34997767

[B98] Tanaka M, Sun F, Li Y, Mooney R (2018) A mesocortical dopamine circuit enables the cultural transmission of vocal behaviour. Nature 563:117–120. 10.1038/s41586-018-0636-7 30333629 PMC6219627

[B99] Thompson RR, Goodson JL, Ruscio MG, Adkins-Regan E (1998) Role of the archistriatal nucleus taeniae in the sexual behavior of male Japanese quail (*Coturnix japonica*): a comparison of function with the medial nucleus of the amygdala in mammals. Brain Behav Evol 51:215–229. 10.1159/000006539 9553694

[B100] Vates GE, Broome BM, Mello V, Nottebohm F (1996) Auditory pathways of caudal telencephalon and their relation to the song system of adult male zebra finches (*Taenopygia guttata*). J Comp Neurol 366:613–642. 10.1002/(SICI)1096-9861(19960318)366:4<613::AID-CNE5>3.0.CO;2-7 8833113

[B101] Veenman CL, Wild JM, Reiner A (1995) Organization of the avian “corticostriatal” projection system: a retrograde and anterograde pathway tracing study in pigeons. J Comp Neurol 354:87–126. 10.1002/cne.903540108 7615877

[B104] Veit L, Nieder A (2013) Abstract rule neurons in the endbrain support intelligent behaviour in corvid songbirds. Nat Commun 4:2878. 10.1038/ncomms3878 24285080

[B102] Veit L, Hartmann K, Nieder A (2014) Neuronal correlates of visual working memory in the corvid endbrain. J Neurosci 34:7778–7786. 10.1523/JNEUROSCI.0612-14.2014 24899702 PMC6608265

[B103] Veit L, Hartmann K, Nieder A (2015a) Spatially tuned neurons in corvid nidopallium caudolaterale signal target position during visual search. Cereb Cortex 27:1103–1112. 10.1093/cercor/bhv299 26656724

[B105] Veit L, Pidpruzhnykova G, Nieder A (2015b) Associative learning rapidly establishes neuronal representations of upcoming behavioral choices in crows. Proc Natl Acad Sci U S A 112:15208–15213. 10.1073/pnas.1509760112 26598669 PMC4679020

[B106] von Eugen K, Tabrik S, Güntürkün O, Ströckens F (2020) A comparative analysis of the dopaminergic innervation of the executive caudal nidopallium in pigeon, chicken, zebra finch, and carrion crow. J Comp Neurol 528:2929–2955. 10.1002/cne.24878 32020608

[B107] Wagener L, Nieder A (2023) Categorical representation of abstract spatial magnitudes in the executive telencephalon of crows. Curr Biol 33:2151–2162.e5. 10.1016/j.cub.2023.04.013 37137309

[B108] Wild JM, Farabaugh SM (1996) Organization of afferent and efferent projections of the nucleus basalis prosencephali in a passerine, *Taeniopygia guttata*. J Comp Neurol 365:306–328. 10.1002/(SICI)1096-9861(19960205)365:2<306::AID-CNE8>3.0.CO;2-9 8822172

[B109] Wild JM, Gaede AH (2016) Second tectofugal pathway in a songbird (*Taeniopygia guttata*) revisited: tectal and lateral pontine projections to the posterior thalamus, thence to the intermediate nidopallium. J Comp Neurol 524:963–985. 10.1002/cne.23886 26287809 PMC4731278

[B110] Wild JM, Krützfeldt NEO (2012) Trigeminal and telencephalic projections to jaw and other upper vocal tract premotor neurons in songbirds: sensorimotor circuitry for beak movements during singing. J Comp Neurol 520:590–605. 10.1002/cne.22752 21858818 PMC3935800

[B111] Yuan RC, Bottjer SW (2020) Multidimensional tuning in motor cortical neurons during active behavior. eNeuro 7:1–24. 10.1523/ENEURO.0109-20.2020 PMC7396810 32661067

[B112] Zeier H, Karten HJ (1971) The archistriatum of the pigeon: organization of the afferent and efferent connections. Brain Res 31:313–326. 10.1016/0006-8993(71)90185-5 5569153

[B113] Zemel BM, Nevue AA, Tavares LES, Dagostin A, Lovell PV, Jin DZ, Mello CV (2023) Motor cortex analogue neurons in songbirds utilize Kv3 channels to generate ultranarrow spikes. Elife 12:e81992. 10.7554/eLife.81992 37158590 PMC10241522

[B114] Zhao K, Nie J, Yang L, Liu X, Shang Z, Wan H (2019) Hippocampus-nidopallium caudolaterale interactions exist in the goal-directed behavior of pigeon. Brain Res Bull 153:257–265. 10.1016/j.brainresbull.2019.09.005 31541677

